# Early Life Programming of Adipose Tissue Remodeling and Browning Capacity by Micronutrients and Bioactive Compounds as a Potential Anti-Obesity Strategy

**DOI:** 10.3390/cells13100870

**Published:** 2024-05-18

**Authors:** M. Luisa Bonet, Joan Ribot, Juana Sánchez, Andreu Palou, Catalina Picó

**Affiliations:** 1 Laboratory of Molecular Biology, Nutrition and Biotechnology (Group of Nutrigenomics, Biomarkers and Risk Evaluation), University of the Balearic Islands, 07122 Palma, Spain; luisabonet@uib.es (M.L.B.); joana.sanchez@uib.es (J.S.); andreu.palou@uib.es (A.P.); cati.pico@uib.es (C.P.); 2 Health Research Institute of the Balearic Islands (IdISBa), 07010 Palma, Spain; 3 CIBER de Fisiopatología de la Obesidad y Nutrición (CIBEROBN), 28029 Madrid, Spain; 4 Artificial Intelligence Research Institute of the Balearic Islands (IAIB), University of the Balearic Islands, 07122 Palma, Spain

**Keywords:** metabolic programming, gestation, lactation, leptin, vitamin A, nicotinamide riboside, polyphenols, polyunsaturated fatty acids, myo-inositol, milk miRNAs

## Abstract

The early stages of life, especially the period from conception to two years, are crucial for shaping metabolic health and the risk of obesity in adulthood. Adipose tissue (AT) plays a crucial role in regulating energy homeostasis and metabolism, and brown AT (BAT) and the browning of white AT (WAT) are promising targets for combating weight gain. Nutritional factors during prenatal and early postnatal stages can influence the development of AT, affecting the likelihood of obesity later on. This narrative review focuses on the nutritional programming of AT features. Research conducted across various animal models with diverse interventions has provided insights into the effects of specific compounds on AT development and function, influencing the development of crucial structures and neuroendocrine circuits responsible for energy balance. The hormone leptin has been identified as an essential nutrient during lactation for healthy metabolic programming against obesity development in adults. Studies have also highlighted that maternal supplementation with polyunsaturated fatty acids (PUFAs), vitamin A, nicotinamide riboside, and polyphenols during pregnancy and lactation, as well as offspring supplementation with myo-inositol, vitamin A, nicotinamide riboside, and resveratrol during the suckling period, can impact AT features and long-term health outcomes and help understand predisposition to obesity later in life.

## 1. Introduction

Obesity is an urgent public health issue worldwide. According to the World Health Organization, in 2022, one in eight people were living with obesity [1]. Obesity is associated with multiple health problems, such as type 2 diabetes mellitus, hypertension, cerebrovascular disease, kidney disease, many types of cancers, and a variety of musculoskeletal illnesses [2,3,4]. Traditional treatments, namely changing dietary habits and increasing physical activity, are hard to implement and often ineffective, as weight regain is common. New gut–brain axis knowledge and recent pharmacological developments have revolutionized the way in how to treat obesity [5], but recurrent weight recovery after any weight loss continues to be the biggest problem [6,7]. Preventive strategies are, therefore, in the spotlight.

In this context, increasing evidence indicates that, besides genetics and contemporary environmental exposures, early life exposures, particularly nutritional cues in critical developmental periods, may have a significant impact on metabolic health and susceptibility to obesity in adulthood. This phenomenon is known as “programming” or “metabolic programming” [8,9,10]. Nutrition in utero and during the early postnatal period, commonly referred to as the first 1000 days from conception to 2 years, is critical. Certain nutritional conditions at these stages typically program an increased propensity toward obesity. In contrast, others may program a relative resistance to it or confer resistance to the “malprogramming” effects of adverse maternal exposures. The mechanisms of metabolic programming have primarily been studied in preclinical rodent models and are interrelated. They include epigenetic effects and effects on telomere length that shape the fate of resident progenitor cells in tissues and modulate cellular senescence, tissue cellularity, and metabolic capacities [11,12,13]; effects on the development of structures and neuroendocrine circuits that are crucial to the control of energy balance, such as regulatory brain centers and corresponding efferent and afferent pathways [10]; and effects on the developing gut microbiome [14], among others. Dietary cues can instruct all of these mechanisms.

Biochemical processes involved in the control of body weight and body adiposity can be programmed. Here, we will focus on the nutritional programming of adipose tissue (AT) features. Obesity is a chronic complex disease defined by the excessive accumulation of fat deposits in AT that can impair health. The defining organ of obesity is thus AT. Mammalian AT displays anatomical, physiological, and functional diversity [15]. Further, AT depots and adipocytes have different origins and metabolic capacities [16]. Taken as a whole, AT can be viewed as an organ that plays a crucial role in regulating whole-body energy homeostasis and metabolism by being able to store lipids, mobilize lipids, produce and secrete signals, and burn fuel substrates to dissipate energy as heat (thermogenesis), all in a regulated manner [17,18]. It is a highly plastic organ that can experience cellular and metabolic remodeling, to different degrees, to allow for its expansion or shrinkage depending on the energy status and other biological signals.

The term “AT remodeling” usually refers to changes in the size, number, or turnover of adipocytes and the renovation of the AT extracellular matrix in response to requirements for tissue growth and expansion, hormones, aging, or pathologies [19,20]. Cell turnover encompasses the processes of cell proliferation, differentiation, and apoptosis, which collectively regulate the overall cell population within the tissue [21]. AT comprises mature adipocytes and a stromal vascular fraction that contains mesenchymal progenitor/stem cells, preadipocytes (not yet differentiated but already committed to becoming adipocytes), endothelial cells, and immune cells. Remodeling involves quantitative and qualitative changes in the stromal vascular fraction cells besides the adipocytes. AT expansion in obesity can be mediated by hypertrophy (enlarged adipocytes), hyperplasia (increased numbers of adipocytes), or both. Expansion through hyperplasia involves the proliferation of progenitor cells or preadipocytes followed by adipogenic differentiation (adipogenesis). It is generally considered a healthier form of expansion that may prevent metabolic dysfunction compared to expansion by adipocyte hypertrophy [22].

Intertwined with the “cellular” facet of AT remodeling, there is a “metabolic” facet, which refers mainly to changes in the capacity of fat depots for substrate oxidative metabolism and thermogenesis. In this context, the distinction between white and brown AT (WAT and BAT, respectively) and the concept of WAT browning or beigeing needs to be introduced. WAT specializes in energy storage and release and is composed primarily of white adipocytes, while BAT specializes in regulated heat production (adaptive thermogenesis) and is composed primarily of brown adipocytes. WAT and BAT also have distinct endocrine functions [23]. The key to BAT thermogenic function is the high oxidative capacity, mitochondrial content, and characteristic expression of uncoupling protein 1 (UCP1) in brown adipocytes. UCP1 is an inner mitochondrial membrane protein that can uncouple cellular respiration from ATP synthesis by facilitating proton re-entry into the mitochondrial matrix, thus circumventing ATP synthase. BAT thermogenesis is mainly controlled by the sympathetic nervous system (SNS) and its neurotransmitter noradrenaline and is influenced by numerous hormonal signals. WAT browning or beigeing refers to the emergence of thermogenically competent, UCP1-expressing cells in WAT depots. These cells, called brite or beige adipocytes, have a distinct origin from brown adipocytes. Evidence suggests they may originate from (Myf5−) progenitor cells resident in WAT depots through de novo differentiation (so-called beige adipogenesis, favored under certain conditions) or through transdifferentiation from pre-existing mature white adipocytes [24]. In rodents, many signals and nutritional stimuli that activate BAT thermogenesis also favor WAT beigeing [25,26]. Because activated BAT and beige WAT can dissipate energy as heat and actively take up glucose and fatty acids from the circulation to serve as fuel, their activation might be protective against obesity and metabolic complications such as diabetes and dyslipidemia. In fact, in humans, the presence of active BAT is associated with cardiometabolic health [27].

**Figure 1:**
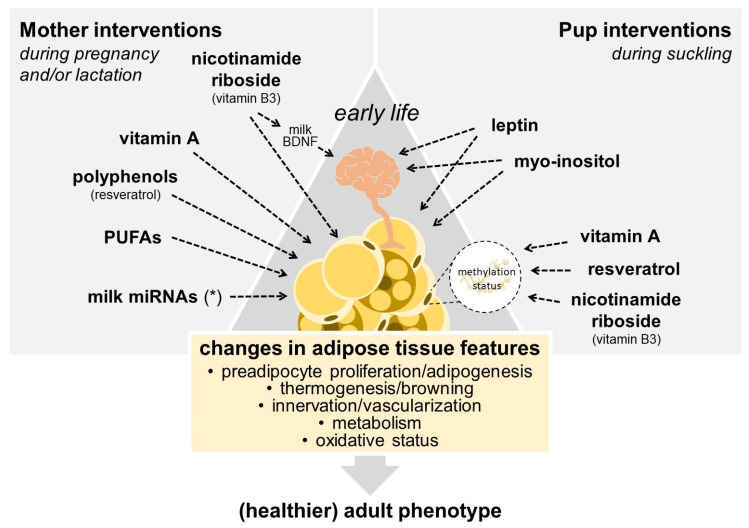
Early life exposures, particularly nutritional cues in critical developmental periods, may have a significant impact on metabolic health and susceptibility to obesity in adulthood. Animal studies reviewed show that mother supplementation with polyunsaturated fatty acids (PUFAs), vitamin A, nicotinamide riboside, and polyphenols during pregnancy and lactation, and offspring supplementation during suckling with leptin, myo-inositol, vitamin A, nicotinamide riboside, and resveratrol, impact adipose tissue features by conditioning local epigenetic modifications and the development of structures and neuroendocrine circuits that are crucial to the control of energy balance, or even other metabolic tissues, with implications for the propensity to obesity later in life. (*) miRNAs in the figure are not interventions in mothers but refer to the miRNAs present in breast milk that are affected by the maternal metabolic status and/or diet and may target adipose tissue features.

Nutritional factors during prenatal and early stages of postnatal development can target both facets of remodeling in the developing WAT and BAT depots, with implications for a propensity to obesity later in life [28,29,30]. Maternal total energy intake (under and overnutrition) and diet macronutrient composition have been extensively researched in this context. For instance, maternal obesity and a high-fat diet (HFD) predispose the offspring to obesity and metabolic disease, and studies in mice and rats showed that maternal HFD or a cafeteria diet during lactation negatively affects the thermogenic function of BAT in the long-term in the offspring [31,32,33]. Instead, this narrative review focuses on specific nutritional compounds whose intake levels at critical developmental periods can program AT features such as cellularity and metabolism in the offspring according to existing evidence, coming mainly from animal studies. The main focus is on compounds that may program obesity resistance or help counteract “malprogramming” caused by adverse maternal conditions through effects on AT. Many of the studies reviewed concentrate on the lactation period, which in rodents is a period of intense adipogenesis in AT, especially in WAT (BAT develops mainly before birth), and hence a vital programming window for AT development [28]. Compounds reviewed include certain micronutrients (vitamins), bioactive compounds, fatty acids, peptide hormones (leptin), and miRNAs present at variable levels in breast milk (Figure 1). Hormones and miRNAs in milk are part of the mechanisms by which maternal dietary conditions exert programming effects on the offspring. Still, they can also be approached and assayed in newborns as nutritional exposures susceptible to manipulation and are thus considered here.

## 2. Nutritional Compounds Active in the Programming of AT

### 2.1. Nicotinamide Riboside

Nicotinamide riboside (NR), a derivative of Vitamin B3 (niacin), is present in breast milk in amounts related to maternal status and intake [34,35,36]. NR is a precursor of NAD^+^ and shows, among others, anti-obesogenic effects [37] and metabolic benefits when administered to adult animals [38]. Increased fat-free mass and decreased fat mass were reported in humans supplemented with NR, resulting in improved body composition while the body weight remained unchanged. [39]. These beneficial effects have been related in rodents to the stimulation of mitochondrial oxidative metabolism in AT and other tissues due to the ability of NR to function as an activator of the NAD^+^-dependent protein deacetylase, sirtuin 1 (SIRT1) [37,40,41].

The programming activity of NR in early life stages has been much less studied [42,43,44,45,46]. Ear and colleagues reported that the dietary NR supplementation of lactating mothers (~24 mg NR per mother per day) increases lactation and milk bioactive content in mice, which is linked to expanded mammary gland biosynthetic capacities [42]. This resulted in persistent physical–including lower body fat–neurobehavioral and neurodevelopmental advantages to their adult offspring [42]. They reported increases in NAD metabolites and the brain-derived neurotrophic factor (BDNF), a growth factor that enhances brain development, in the milk of dietary NR-supplemented lactating mothers. This could explain the observed long-lasting neurodevelopmental benefits to their offspring, including an enhanced special memory and hippocampal neurogenesis. Importantly, although fat browning was not addressed in the study by Ear and colleagues, BDNF can act centrally to induce a brown fat gene program in WAT [47].

Direct oral NR supplementation to the pups during the suckling period elicited programming effects on AT cellular and molecular features [43,44,45]. The supplemental dose eliciting programming effects was very low (24 μg to 45 μg NR per pup per day, increasing from postnatal day 2 to day 20). NR supplementation during suckling led to an enhanced thermogenic and catabolic gene expression profile in the BAT and subcutaneous WAT after a 10-week obesogenic HFD challenge starting in adulthood (at three months of age). Concomitantly, it led to improved metabolic responses, such as a lower leptin/adiponectin ratio and reduced lipolysis compared to control mice on the HFD [43]. Interestingly, all these effects were observed in males and were not present in females, for which supplementation was without effect or even had opposite effects (to those observed in males).

Mechanisms behind the sex-dependent programming of the beige adipose thermogenic/oxidative phenotype by neonatal NR supplementation likely include changes in the WAT-resident progenitor cells of young mice modifying their transcriptional program toward an increased commitment to beige (versus white) adipogenesis, as suggested by results in primary preadipocyte cultures [44]. In particular, the expression of genes related to browning (such as *Ucp1*, *Prdm16*, *Slc27a1*) was increased in the primary cultures derived from the subcutaneous WAT of recently weaned (35-day-old) male mice supplemented with NR, even though the depot of origin lacked an obvious brown fat-like gene expression signature. In the primary cultures derived from the WAT of NR-supplemented females, the expression of brown/beige markers was unaffected or was even decreased (for *Ppargc1b*, *Tmem26*, *Hoxc9*) [44]. The turnover of adipocytes in AT occurs throughout life from the progenitor cells/preadipocytes residing in the fatty deposits [48], making these cells natural depositors of programming information from dietary cues in critical life stages.

Long-term DNA methylation modifications may contribute to the programming of an up-regulated expression of browning-related genes such as *Slc27a1* and *Prdm16* in subcutaneous WAT, brought about by the mild supplementation of NR during the suckling period in male mice [45]. Thus, neonatal NR-supplementation resulted in the hypomethylation of specific CpG sites in the distal promoter and intragenic regions of the *Slc27a1* gene and the *Prdm16* gene proximal promoter, and it counteracted the HFD-induced changes in other methylation marks of these genes in WAT in adulthood. Methylation changes at CpGs close to putative binding sites for transcription factors (TF) known to be relevant in the context of AT and energy metabolism (NRF1, PPARγ) were observed, which could modulate TF binding and, thus, transcription [45]. Interestingly, NR exerted down-regulatory effects on the gene expression of main de novo DNA methyltransferases both in the subcutaneous WAT of young mice supplemented with NR during suckling and in 3T3-L1 adipocytes in culture [45]. This suggests one mechanism that, in the context of the persistence of the affected epigenetic marks, could explain the hypomethylation of DNA sites in WAT as observed in adulthood. How site-specific hypomethylation is achieved remains unclear.

Secondary to the programming of more oxidative adipose tissues (or as a consequence of NR epigenetic effects on other tissues), male mice supplemented with NR during suckling were protected against HFD-induced triacylglycerol accumulation in skeletal muscle and displayed lower triacylglycerol levels, a degree of steatosis in the liver, distinct capacities for fat oxidation and decreased lipogenesis in both tissues. These phenotypic differences paralleled signs of enhanced SIRT1 and AMP-dependent protein kinase signaling in these tissues [46].

### 2.2. Vitamin A

Vitamin A (VA) is an essential micronutrient with two dietary sources: preformed VA (retinol and retinyl ester) derived from animal sources and provitamin A carotenoids (mainly β-carotene and β-cryptoxanthin) derived from colorful fruits and vegetables. The acidic form of VA, retinoic acid (RA), is a potent regulator of mammalian gene expression by serving as an activating ligand of the transcription factors of the nuclear receptor superfamily, the retinoid receptors (RARs), and other mechanisms [49]. Retinoids comprise VA (retinol) and its metabolites, such as retinaldehyde and RA.

VA, primarily as RA, modulates key aspects of adipocyte and AT biology, including adipogenic differentiation (adipogenesis), hypertrophic expansion, the capacity for fat oxidation and thermogenesis, and the secretory function, with possible benefits in the context of controlling body fat, obesity and its comorbidities [50,51,52,53]. Dietary VA supplementation, dietary β-carotene conversion to RA, and direct RA treatment have all been demonstrated to exert anti-obesity effects in animal models [54,55,56,57,58,59]. Recent evidence indicates that local β-carotene conversion to RA in the AT can contribute to body fat reduction [60]. The anti-adiposity action of RA is attributed to its ability to (i) induce UCP1 expression and stimulate both BAT thermogenesis [61,62,63] and WAT browning [64,65]; (ii) decrease the levels and activity of the master proadipogenic/pro-lipogenic transcription factor peroxisome proliferator-activated receptor γ (PPARγ) in the WAT [57,66]; and (iii) stimulate fatty acid oxidation in non-adipose tissues [67,68,69]. In humans, β-carotene in plasma [70] and dietary VA intake [71] are inversely associated with adiposity, and anti-obesity effects of β-carotene-rich carotenoid supplements have been described [72].

The accumulated evidence linking VA to beneficial effects regarding the control of body fat reserves has prompted pre-clinical research on the programming activity against the obesity of supplemental VA in early life. These studies need to consider the toxicity and teratogenicity of VA. This vitamin and its metabolite, RA, play key roles in fetal morphogenesis and organ development [73]. VA requirements during pregnancy are increased, yet VA at high supplementation levels can exert teratogenic effects in animals and humans [74]. The WHO considers a dose of up to 10,000 IU daily or 25,000 IU weekly safe during pregnancy [75], a threshold about five times higher than the Population Reference Intake (PRI) for VA in pregnant women (700 µg/day or 2331 IU/day) [76]. Therefore, there is limited room for research on the impact of VA at levels moderately above normal intake on fetal development and its long-term consequences. The PRI for VA in lactating women is 1300 µg/day or 4329 IU/day [76]. Children are born with short VA reserves, and human milk provides both preformed VA (retinol and retinyl esters) and provitamin A carotenoids to fulfill these requirements [77]. Interestingly, carotenoids are readily detected in breast milk but are often lacking in formula milk [78,79]. Furthermore, breast milk provitamin A carotenoid concentrations are decreased in obese mothers [80,81]. It is conceivable, therefore, that a deficit in provitamin A supply contributes to a relative “malprogramming” in formula-fed infants and infants of mothers with obesity, prompting research on the effects of mild excess VA supply during suckling/lactation.

Retinoids inhibit adipogenesis in cell models [82] and in vivo in adult mice exposed to a high-fat diet [59]. In line with an antiadipogenic action of retinoids, in rats, a 3-fold excess supplemental VA as retinyl palmitate during suckling triggers changes in the developing WAT that result in smaller adipocytes with lower expression levels of PPARγ and higher proliferating cell nuclear antigen (PCNA) at weaning [83]. The increased proliferative state—attributed to an increased number of immature adipocytes rather than preadipocytes since preadipocyte marker genes (*Pref1*, *Sox9*, *Klf2*) were not upregulated—potentiated WAT hyperplasic expansion in the face of an obesogenic diet. Thus, when fed a high-fat diet, the VA-supplemented rats gained the same body weight but higher adiposity and WAT DNA content than control rats. Notably, the increase (30%) in adiposity did not correlate in these animals with a worsening of metabolic complications related to obesity. In the same direction, a 4-fold excess dietary VA (as retinol in food) led to higher adiposity gain in three-week-old rat weanlings challenged with a cafeteria diet for eight days, which paralleled a higher proliferation competence of the adipocyte precursor cells isolated from the animals’ fat depots [84]. Further, adipocyte hyperplasia and a smaller adipocyte size were found in fat depots of lambs under standard feed supplemented with a 30-fold excess VA as retinyl palmitate from birth until the end of the growth period, despite no effects on total body adiposity [85]. Overall, these studies show that VA supplementation in early postnatal life can favor adipocyte hyperplasia, perhaps as a consequence of inhibiting the differentiation of fully mature adipose cells. This effect is interesting since adipocyte hyperplasia has a protective impact on metabolic dysfunction induced by excessive caloric intake. An insufficient number of adipocytes favors adipocyte hypertrophy, hypoxia, and inflammation, a key cause of metabolic dysfunction [22].

Mild VA supplementation during gestation and lactation (at twice the normal intake as retinyl acetate, in drinking water) also favored adipocyte hyperplasia in the offspring, as indicated by increased white and brown fat mass, smaller adipocyte size, and an elevated expression of both preadipocyte genes and mature adipocyte markers in fat depots at weaning [86]. However, this supplementation also strongly upregulated beige/brown adipogenesis in both WAT and BAT. This was indicated by an increased number of UCP1-positive multilocular adipocytes in subcutaneous WAT, a higher expression of *Ucp1* and other brown adipose genes in WAT and BAT, and a higher core body temperature at weaning. As a result, the offspring of VA-supplemented dams were efficiently protected from obesity and metabolic dysfunction when challenged for 5 months with a high-fat diet starting at 30 days of age [86]. The intraperitoneal injection of RA during pregnancy (at embryonic days E10.5 and E13.5) reproduced the effects on adipocyte hyperplasia and beige/brown adipogenesis in weanling offspring. The effects of maternal retinoids during the fetal stage were shown to be RAR-mediated and related to the promotion of angiogenesis in the fetal ATs, leading to an expanded population of PDGFRα^+^ adipose progenitor cells. These are proliferative cells capable of both white and beige adipogenesis [87], and therefore their expansion could explain both white adipocyte hyperplasia and increased beige adipogenesis.

The timing of VA supplementation during development may thus critically condition the consequences on offspring adiposity. VA supplementation promotes AT hyperplasia in the offspring when administered to pregnant and lactating dams [86], during pregnancy only [86], or directly to suckling or recently weaned animals [83,84,85], albeit not necessarily by the same mechanisms, while the stimulation of beige/brown adipogenesis apparently requires VA supplementation during fetal life [86]. Notwithstanding other differences in the study designs, it is worth noting that in mice, brown fat fully develops, and white fat begins to develop during the fetal stage [88,89]. Therefore, it is likely that many cells acquire the commitment to brown/beige adipocytes during fetal life.

Recent studies indicate that VA supplementation in early life may ameliorate malprogramming caused by an inadequate maternal diet. In particular, in rats, a 50-fold excess VA as retinyl acetate in the maternal diet during lactation reduced excess adiposity and other harmful effects caused by maternal high-fat diet feeding during pregnancy and lactation in the offspring studied up to postnatal day 35 [90]. Oral RA treatment to the suckling pups (4 µg/g body weight, every three days from postnatal day 5 to 20) also ameliorated the excess adiposity in neonates born to high-fat diet-fed dams, studied up to postnatal day 20 [91]. These works have studied the short-term consequences (in newborns or recently weaned animals) of VA supplementation at relatively high doses. Our recent (unpublished) results indicate that mild-direct VA supplementation as retinyl palmitate to mouse pups at a physiologically relevant level (3-fold excess) during suckling can lessen the worsening in glucose control, associated with maternal and lifelong obesogenic Western diet feeding studied at 4 months of age.

Provitamin A carotenoids such as β-carotene are generally considered to have lower toxicity and teratogenicity than preformed VA and may, therefore, represent a safer form of VA provision. However, β-carotene is not only a precursor to VA; it has VA-independent effects and can be metabolized to other apocarotenoids besides the VA retinoids. Little is known about tissue β-carotene metabolism in the fetal and neonatal periods [92] and the eventual metabolic programming effects of β-carotene. We have shown that supplemental oral β-carotene at a mild dose is partly absorbed intact by suckling rats (so that it accumulates in the serum and liver) and is partly metabolized to retinoids (leading to increased levels in the liver and WAT after supplementation) [93]. Unlike supplementation with retinyl ester, neonatal supplementation with an equivalent dose (3-fold excess) of VA in the form of β-carotene did not affect adipocyte size, PPARγ, or PCNA expression in WAT at weaning [93]. Moreover, retinyl palmitate and β-carotene supplementations differentially impacted epigenetic methylation marks in the WAT of rats at weaning in these and other genes related to AT development and function [94]. The hypermethylation of the *Pparg* promoter and hypomethylation of the *Pcna* promoter were observed, together with inverse changes in mRNA levels, in the retinyl palmitate-treated group only. In a recent study, maternal β-carotene supplementation during pregnancy (leading to a 1.57-fold increase in retinol content in the maternal liver) was shown to increase visceral fat weight and PPARγ expression and cause metabolic impairments in the adult female offspring [95]. Overall, studies suggest that supplementing preformed VA or β-carotene in early life may entail different outcomes regarding adiposity programming, which deserves further investigation.

### 2.3. Resveratrol and Other Polyphenols

Several reports have indicated beneficial metabolic programming effects of maternal supplementation with polyphenols, including resveratrol, during the fetal and lactation stages, especially in the context of maternal HFD/obesity, as well as a reduction of body fat accumulation and metabolic disorders, such as hypertension, insulin resistance, and inflammation, and cognitive impairment, in the offspring in adulthood (reviewed in [96,97,98]). Since polyphenols have a wide range of activities, their beneficial effects are attributed to several interrelated mechanisms affecting oxidative stress, nutrient-sensing signals, and others [99]. In addition, polyphenols reverse adverse epigenetic regulation by, among others, altering DNA methylation or histone modification [100].

Focusing on anti-obesity action and AT direct effects, a polyphenol of reference is resveratrol (3,5,4′ trihydroxy-trans-stilbene), a polyphenol produced by and present in plant species such as blackberries, peanuts, and grapes. Resveratrol shows anti-obesogenic effects and metabolic benefits when administered to adult animals, which have been related to the stimulation of the mitochondrial oxidative metabolism in AT, among other tissues [101,102,103]. Although the results are more controversial, interesting effects of resveratrol supplementation have also been demonstrated in humans [104]. Apart from its effects as a direct antioxidant agent or through indirectly upregulating the expression of antioxidant defensive enzymes [105], resveratrol has a stimulatory effect on cellular oxidative metabolism that has been related to its ability to activate SIRT1, which is a crucial regulator of mitochondrial oxidative metabolism [106]. Also related to SIRT1 activation [107], another biological activity of resveratrol that may be relevant in the context of metabolic programming is its capacity to augment telomerase activity and contribute to telomere maintenance, as demonstrated in endothelial progenitor cells [108].

Regarding programming effects, animal studies have shown that the maternal intake of different polyphenols, including resveratrol, during pregnancy and/or lactation can favorably program the offspring towards a better inflammatory profile under conditions of HFD feeding, including a less proinflammatory gene expression signature in AT [97,109], which could be protective against obesity-related metabolic complications. Additionally, resveratrol supplementation to HFD-fed mouse dams during pregnancy and lactation (~4 mg per mother per day) promotes BAT metabolic activity and the browning of subcutaneous WAT in their male offspring, conferring protection against HFD-induced obesity and insulin resistance in adulthood [110]. This was mediated by an increased expression of PRDM16 and the activation of the SIRT1/AMPK pathway in BAT and WAT at weaning [110]. Other studies using the HFD-obese rat model highlighted that maternal resveratrol supplementation has a reprogramming role for progeny involving the upregulation of SIRT1 in WAT [111]. Maternal resveratrol supplementation (~10 mg per mother and day) only during lactation also attenuated body weight and plasma and liver triacylglycerol levels in adult male rat offspring and programmed enhanced AMPK activation through SIRT1 upregulation in the adult liver (effects on AT were not reported) [112]. The programming effects of maternal resveratrol intake are different in males and females, although most studies reported on male offspring only [98]. It is worth mentioning that, even though health benefits are widely reported, some studies pointed to concern about the adverse effects of maternal polyphenols rich-extracts intake during pregnancy and/or lactation [113].

The biological effects of maternal polyphenol supplementation on programming are attributable to both effects on the mother and directly on the offspring. Resveratrol most likely alters maternal metabolism, which could change the intrauterine and early postnatal nutritional environments, including potential changes in breast milk macronutrient and micronutrient composition and milk microbiota, beyond the possible presence of resveratrol and its metabolites (presumable but not reported in those studies). However, since resveratrol can cross the placenta as other polyphenols, direct effects on the fetus cannot be discarded [114,115].

Interestingly, the direct oral supplementation of mouse pups with a low dose of resveratrol (~3 μg to ~16 μg per pup per day) during suckling has sex-specific programming effects on AT features [43,44,45]. This supplementation increased the expression of genes related to browning, oxidative metabolism, and mitochondrial biogenesis and function in the subcutaneous WAT of adult male mice but not in females [43]. The browning-promoting effect was confirmed with the increase in the content of mitochondrial DNA and the presence of multilocular adipocytes positive for UCP1 and COXIV immunostaining in the subcutaneous WAT of adult mice. Along with WAT browning, adult male mice treated with resveratrol during suckling presented a more oxidative metabolic profile in skeletal muscle [116], a delayed body weight gain [43] and blunted triacylglycerol accumulation in skeletal muscle [43], compared to controls on an HFD [43].

Like for NR (Section 2.1), mechanisms behind the sex-dependent programming of the beige AT phenotype through postnatal resveratrol supplementation during suckling likely include changes in WAT-resident progenitor cells toward a greater commitment to beige (versus white) adipogenesis and long-term effects on DNA methylation marks in brown/beige-related genes in subcutaneous WAT. For both treatments, the expression of brown/beige adipocyte marker genes was upregulated in primary cultures established from the WAT of young treated male animals and downregulated in those established from the WAT of treated females [45]. In the case of resveratrol treatment, the widespread downregulation of these genes in the female-derived WAT primary cultures was particularly evident. Both treatments modified methylation marks in *Slc27a1*, *Prdm16* and the HFD-dependent dynamics of these marks in the adult WAT, with distinct and common effects that could contribute to increased transcription. Additionally, both early postnatal treatments affected the gene expression of de novo DNA methyltransferases in the WAT of recently weaned animals. The down-regulation of epigenetic modulators was reported in association with the attenuation of programmed kidney injury by maternal green tea polyphenol supplementation during lactation [117]. Overall, the programming effects of NR and resveratrol supplementation in early life on the beige phenotype are very similar and show a similar sex dependence, as could be expected considering that the two compounds are activators of SIRT1, albeit by different mechanisms [37,118]. However, both compounds have additional biological targets that could be involved in less similar programming effects observed in tissues such as brown fat [43] or the liver [46,116].

Our studies using direct pup supplementation would support that resveratrol is present in the breast milk of resveratrol-supplemented mothers since similar effects on the WAT phenotype are found in both approaches. Although data on breast milk polyphenolic composition and its association with plant-based food intake or food supplementation are limited [119], it has been observed in the vertical transmission of phenolic compounds and their metabolites (both enzymatic and microbial) to offspring via milk in rats fed with extra virgin olive oil [120]. Opening new approaches, a pilot study in humans demonstrates that polyphenols from maternal pomegranate juice consumption are absorbed by the nursing infant from breast milk, excreted in infant urine, and, interestingly, impact the infant gut microbiome [121]. The modulation of the gut microbiome and their derived metabolites can, in turn, impact the brown/beige activity in WAT and BAT [122].

### 2.4. Polyunsaturated Fatty Acids

The polyunsaturated fatty acids (PUFA) linoleic acid (LA; C18:2n-6) and α-linolenic acid (ALA; C18:3n-3), along with their long-chain products arachidonic acid (AA; C20:4n-6), eicosapentaenoic acid (EPA; C20:5n-3), and docosahexaenoic acid (DHA; C22:6n-3), exert significant influences on AT function and development and overall body adiposity.

Fish oil, which is rich in the n-3 PUFA EPA and DHA, may decrease abdominal adiposity in humans [123], and animal studies corroborate an anti-obesogenic effect of EPA/DHA feeding linked to BAT activation and WAT browning (reviewed in [26,124]). Mechanisms explaining the EPA stimulation of brown/beige fat include (i) the activation of free fatty acid receptor 4 (FFAR4, also known as GPR120), leading to the upregulated expression of microRNAs (such as miR-30b and miR-378) promoting brown adipogenesis in adipose precursor cells [125], and the secretion of FGF21 by the adipocytes [126]. FGF21 activates brown/beige adipocytes in a paracrine/autocrine manner and also acts centrally to induce SNS activity [127]; (ii) the stimulation of Neuregulin 4 (NRG4) production by preadipocytes [128], a batokine/adipokine linked to brown/beige adipocyte activation [129,130]; and (iii) the activation of transient receptor potential vanilloid 1 (TRPV1) receptor [131]. TRPV1 is present (among other sites) in the gastrointestinal tract, on vagal afferent fibers to the brain, and its interaction with dietary agonists such as EPA may lead to SNS activation and catecholamine release to BAT/WAT [131]. On the other hand, n-6 PUFA, especially AA, promote white adipogenesis by serving as a precursor of prostacyclin, a signal that stimulates preadipocyte proliferation in a paracrine loop, and by activating PPARs [132]. The n-3 PUFA are not metabolized to prostacyclin and do not promote adipogenesis [133] or even inhibit adipogenesis [134] in cell models.

The composition of dietary fatty acids, especially PUFA, in the perinatal diet influences the early development of AT and adiposity later in life. Animal studies showed that the intake of a high n-6/n-3 PUFA ratio during fetal and postnatal life promotes early AT growth and obesity [135,136]. The maintenance of a Western-like fat diet with a high n-6/n-3 PUFA ratio (LA/ALA of 28) gradually enhances fat mass over generations in mice [137]. In lactating women, breast milk’s fatty acid composition reflects the composition of the maternal diet [138]. Infant exposure to a high n-6/n-3 PUFA ratio during gestation and breastfeeding is associated with increased pediatric adiposity up to 3 years of age [139]. Adipose deposition by four months of age is directly associated with the n-6/n-3 PUFA ratio in human milk independent of maternal BMI [140]. A high n-6/n-3 PUFA ratio in the diet may imply a substantial increase in LA, a decrease in ALA, or both. The n-6/n-3 ratio in Westernized diets has markedly increased since 1960, mainly as a consequence of replacing the saturated fatty acids in processed foods with n-6-rich fatty acid oils, especially soybean oil [141]. The increase in the n-6/n-3 PUFA ratio in modern diets and breast milk in the last 60 years has been postulated to contribute to the current obesity pandemic [135,136].

Contrary to perinatal exposure to high n-6/n-3 PUFA ratios, perinatal exposure to a low endogenous maternal n-6/n-3 PUFA ratio conditions adult resistance to dietary obesity in mice [142]. This was demonstrated in the wild-type offspring of hemizygous dams for the overexpression of the *fat-1* gene from *Caenorhabditis elegans*, which encodes a fatty acid desaturase (absent in mammals) that converts n-6 PUFA to n-3 PUFA. By postnatal day 14, the subcutaneous WAT of wild-type pups receiving low perinatal n-6/n-3 ratios displayed more adipocytes smaller in size, a suppressed adipogenic gene expression (including *Pparγ*), and the hypermethylation of the *Pparγ* promoter compared to the subcutaneous WAT of wild-type pups receiving high perinatal n-6/n-3 ratios [142]. Pups exposed to low endogenous maternal n-6/n-3 PUFA also had elevated circulating adiponectin. However, no changes in beige or brown adipocyte regulators were observed in the subcutaneous WAT of pups. Obesity resistance in these animals as adults was associated with a lower positive energy balance and food intake, greater energy expenditure and lipid fuel substrate preference, and better glucose clearance [142]. Lowering the endogenous maternal n-6/n-3 PUFA ratio also reduced maternal obesity-associated inflammation and limited adverse developmental programming in the wild-type offspring of high-fat diet-fed dams, protecting against excessive body fat accumulation and insulin resistance in adulthood [143].

It is likely that both the excess of n-6 PUFA and paucity of n-3 PUFA in early life stages, characteristic of more Western-style high-fat diets, are involved in the adverse metabolic programming of the offspring. In this context, animal studies indicate the benefits of n-3 PUFA supplementation. Maternal n-3 PUFA supplementation (with 3% PUFA from fish oil) enhanced BAT development in the offspring in mice, leading to the upregulated expression of brown-specific gene and protein profiles at weaning [144]. This paralleled the modulation of epigenetic factors for BAT development, including increased levels of a functional cluster of brown-specific miRNAs (miR-30b, -193b, and -365) and histone acetylation at the promoter/enhancer region of brown adipocyte genes *Ucp1* and *Pgc1a* [144]. Importantly, maternal n-3 PUFA supplementation conferred long-lasting thermogenic benefits to offspring. At 11 weeks of age, the animals were more cold-resistant and had higher energy expenditure (no differences in respiratory exchange ratio) than the controls. After cold exposure, they presented a more active BAT and massive subcutaneous WAT browning compared to the control mice not exposed to maternal n-3 supplementation [144]. It remains to be tested if maternal n-3 supplementation also confers resistance against dietary obesity in adulthood.

Contrary to conventional n-6 PUFA, conjugated linoleic acid (CLA)—which represents the positional and geometric isomers of linoleic acid—has been associated with anti-obesity effects in several animal and human studies and positive effects in the context of metabolic programming. In rats, maternal CLA supplementation (mix of 50% each *c9,t11* and *t10,C12* CLA isomers) to a high-fat diet normalized the inflammatory phenotype in mothers and blunted the adverse early life growth trajectory (including reduced fetal size and accelerated growth in the pre-weaning period) and the impaired insulin sensitivity of offspring at weaning [145]. CLA supplementation during pregnancy/lactation in rats also had long-term benefits for the offspring. It ameliorated the maternal high-fat diet-induced programming of early onset puberty, increased fat mass, increased hyperlipidemia in female offspring [146] and prevented programmed excess adiposity and metabolic impairments in the adult male offspring [147]. The latter effects were associated with a smaller adipocyte size and a decreased expression of the preadipocyte marker *Dlk1* in the retroperitoneal WAT depot, suggesting a healthier AT expansion in the male offspring exposed to maternal CLA supplementation [147]. Those studies did not report the effects of maternal CLA supplementation on BAT development or WAT browning.

### 2.5. miRNAs in Milk

MicroRNAs (miRNAs), non-coding RNAs of about 22 nucleotides, are present in breast milk [148], and research has suggested their bioavailability in newborns [149,150]. Thus, miRNAs supplied by breast milk can allow effective molecular communication between the mother and the infant [151,152], participating in signaling and regulatory mechanisms during the early postnatal period and maybe contributing to metabolic programming. For example, many studies have linked the presence of specific miRNAs in milk to the regulation of immune responses [152,153,154], epigenetic mechanisms [155,156,157], metabolism [153], and also adipogenesis [158].

The implication of miRNAs controlling critical aspects of AT physiology, such as brown and white adipocyte differentiation, proliferation, WAT browning, and adipocyte function, has extensively been described (reviewed in [159,160]). Interestingly, many adipogenesis-related miRNAs are found in milk, increasing the interest regarding their role as modulators of the reduced risk of obesity associated with breastfeeding. Although it is difficult to establish a direct cause–effect relationship between the effects of miRNAs supplied through milk on the AT development of newborns and the metabolic programming of obesity risk, several human studies and animal models have suggested this possibility [161,162,163,164,165,166,167,168,169].

In a cohort of 60 lactating women (half of them with overweight/obesity), six miRNAs known to be involved in adipogenesis and insulin signaling were screened for potential effects on infant growth depending on their levels in milk. Notably, among these miRNAs, miR-148a and miR-30b emerged as noteworthy. An inverse correlation was found between 1-month breast milk miR-148a levels and infant fat mass and weight at one month of age. In contrast, a direct correlation of miR-30b was found [168]. These results were also observed in another study that included 175 women, 35 of them with gestational diabetes mellitus (GDM) [169]. The inverse correlation between 1-month breast milk miR-148a levels and infant weight was also found at six months of age in the latter study [169]. Moreover, miR-148a and miR-30b levels were decreased in breast milk from women with overweight/obesity and GDM [168,169], suggesting that the maternal condition impacts breast milk miRNA levels, affecting metabolic outcomes in offspring.

miR-148a is one of the most abundant miRNAs in milk [170]. The role of miR-148a in adipogenesis has been evidenced in several cell models. miR-148a promotes the adipogenesis of human adipose-derived mesenchymal stem cells by repressing Wnt1, an inhibitor of adipogenesis [171] and of rabbit and ovine pre-adipocytes through the inhibition of PTEN, one of the targets of this miRNA and another inhibitor of adipogenesis [172,173]. In addition, miR-148a inhibits the proliferation of ovine pre-adipocytes [173]. It has also been reported that miR-148a promotes adipogenesis via targeting the Wnt5a/Ror2 pathway in bone marrow mesenchymal stromal cells from rats [174] or silencing Wnt10b mRNA in 3T3-L1 cells [175]. Further, higher miR-148a expression is found in the WAT of diet-induced obese mice and human subjects with obesity [171]. Therefore, there is considerable evidence that miR-148a promotes adipocyte differentiation by different mechanisms.

miR-148a has also been linked to decreased CpG island methylation at crucial developmental gene promoters by suppressing DNA methyltransferase [156]. Melnik and coauthors proposed a hypothesis of the putative impact of miR-148a in milk exosomes in the epigenetic modulation of WAT and BAT [156,157]. On the one hand, miR-148a could stimulate adipogenesis by promoting the hypomethylation of FTO, thus promoting the expression of FTO and its downstream target C/EBPɑ, an early adipogenic transcription factor. Additionally, miRNA-148a could directly target AMPK, SIK1, and WNT10B mRNAs, thus attenuating the inhibitory action of these targets on mTORC1, SREBP1c, and PPARγ signaling pathway, respectively [156]. On the other hand, the suppression of DNA methyltransferase by miR-148a could result in the demethylation of the UCP1 gene enhancer/promoter, thus increasing UCP1 expression, thermogenesis, and the conversion of white to beige/brown adipocytes [157]. There is experimental evidence for the regulation of UCP1 gene expression in murine AT through changes in the methylation status of its enhancer/promoter [176]. However, the experimental evidence of a modulatory role of miR-148a on brown/beige adipogenesis is lacking, to the best of our knowledge.

Regarding miR-30b, it has been described to promote brown and beige adipogenesis [160]. miR-30b expression increased during brown adipocyte differentiation and in AT following cold exposure or β-adrenergic stimulation [177]. Moreover, miR-30b overexpression induced the expression of thermogenic genes such as *Ucp1* and *Cidea* in brown adipocytes and adipocytes derived from subcutaneous WAT, while miR-30b knockdown suppressed the expression of these genes [177]. A molecular mechanism explaining these effects involves the receptor-interacting protein 140 (RIP140). This protein represses the UCP1 gene enhancer/promoter and is a target of miR-30b [177]. Thus, miR-30b could positively regulate brown and beige adipogenesis by downregulating RIP140. The impact of breast milk miR-30b on thermogenesis or browning in lactating infants remains unknown.

In a small cohort of lactating women involving 38 women with normal-weight and 21 with overweight/obesity, our group has described inverse associations between the levels of miR-103, miR-17, miR-181a, miR-222, miR-let7c, and miR-146b in breast milk and the BMI of their children at two years of age in the normal-weight mothers that, interestingly, were lost in the mothers with obesity [162]. All these miRNAs have been associated with some extent with AT development and function, as explained next. In several cell models, **miR-103** has proven to be a pro-adipogenic miRNA [160]. In porcine preadipocytes in culture, miR-103 induced adipogenesis by targeting retinoic acid-induced protein 14 (RAI14), a protein whose expression normally declines during differentiation [178]. In murine preadipocytes, miR-103 increased *Pparg* expression and promoted adipogenesis by targeting the anti-adipogenic transcription factor myocyte enhancer factor 2D (MEF2D) and activating the AKT/mTOR pathway [179]. miR-103 also promotes apoptosis in preadipocytes by targeting WNT family member 3a (Wnt3a) [180], suggesting that miR-103 could be related to increased adipocyte apoptosis found in the WAT of subjects with obesity [181]. Moreover, miR-103 negatively regulates insulin sensitivity by targeting caveolin-1, a modulator of the insulin receptor [182]. Studies in obese human AT show that miR-103 is upregulated (reviewed in [183]). On the contrary, the activity of **miR-17a** improves insulin resistance, and its levels are reduced in WAT in obesity [160]. miR-17 targets Ask1 (apoptosis signal-regulating kinase 1), a MAP kinase family member involved in the regulation of macrophage activation; the downregulation of Ask1 by miR-17 could thus contribute to the prevention of macrophage-mediated inflammation in the AT, improving insulin resistance [184]. **miR-181a** promotes adipogenesis by targeting TNF-α [185]. **miR-222** levels are increased in the WAT of diet-induced obese mice [186] and type 2 diabetic rats [187]. Increased circulating levels have been detected in adults and children with obesity and T2D patients [188,189,190]. The transcriptome analysis of preadipocytes transfected with a mimetic of miR-222 showed inhibition in metabolic pathways related to insulin signaling, adipogenesis, and lipid metabolism [164]. Overall, these results suggest that miR-222 can repress the insulin signaling pathway in WAT and participate in developing insulin resistance. **miR-let7c** belongs to the let-7 family. Let-7 are key miRNAs, which have been reported to regulate adipogenesis [160]. Although miR-let7c has not been directly related to the modulation of AT, in humans, circulating let-7c is associated with changes in lipid profile and insulin resistance [191]. Finally, **miR-146b** appears to promote adipogenesis since its levels increased during the adipogenic differentiation of 3T3-L1 cells, and its inhibition decreased the differentiation of these cells into adipocytes [192]. The pro-adipogenic activity of miR-146b appears to involve suppressing Sirtuin 1 and Sirtuin 1 downstream effects [192]. miR-146b has also been related to WAT browning, as the aging-dependent inhibition of cold-induced perivascular adipocyte browning is accompanied by decreased miR-146b levels [193]. Therefore, the maternal obesity-associated deregulation of these milk miRNAs with a putative role in AT development could influence perinatal AT imprinting, potentially contributing to the higher BMI in their children.

Maternal diet can change the milk miRNA profile. In animal models, we have verified that the maternal intake of an obesogenic diet during lactation affects specific miRNA levels in milk [161]. Higher levels of miR-222 and lower of miR-200a and miR-26a were found in milk of rats fed the obesogenic diet [161]. These changes could be related to adverse metabolic effects observed in the progeny. Specifically, the offspring of rats fed a cafeteria diet during lactation presented a higher fat mass and metabolic features of the thin-outside-fat-inside phenotype [194,195]. We have indirect evidence of how changes in these miRNA levels in milk could contribute to the higher adiposity in the offspring. A general decrease in the expression of key genes related to insulin signaling, adipogenesis, and lipid metabolism was observed in 3T3-L1 cells transfected with a miR-222 mimetic [164]. The transfection with a mimetic of miR-26a in 3T3-L1 cells decreased the expression of *Pten*, *Hmga1*, *Stk11*, *Rb1,* and *Adam17*, a set of putative target genes of this miRNA related to the development of AT [165]. Interestingly, the offspring of dams fed the obesogenic diet, breastfed with lower levels of miR-26a, and showed a higher expression of *Hmag1*, *Rb1*, and *Adam17* in retroperitoneal WAT compared to controls [165]. Notably, elevated levels of miR-222 in the mammary gland (at weaning) and in milk (on day 15 of lactation) were also observed in rat dams that were fed a Western diet from one month before gestation until the end of lactation [196]. This dietary condition was also associated with adverse programming effects on offspring [197]. Interestingly, the implementation of a healthy diet during lactation, which mitigated most of the detrimental programming effects in the offspring [197], also normalized the expression levels of miRNA-222 in both the mammary glands and milk to values of control rats [196].

In another study, the maternal dietary supplementation of leucine, betaine, and oleic acid during lactation in rats reduced the levels of specific miRNAs in milk (miR-222, miR-27a, miR-103, and miR-200a). These changes could be related to metabolic alterations observed in the offspring, which displayed increased body fat and HOMA-IR. Body fat content in adulthood was negatively associated with the levels of these miRNAs in milk [163].

Overall, maternal factors, including diet and metabolic health status (obesity or gestational diabetes), can modify the composition of specific miRNAs in breast milk. While other factors may be involved, there is increasing evidence that suggests that miRNAs supplied through breast milk could contribute to mother–baby communication, potentially modulating the early metabolic programming of AT development. This modulation could occur through the direct regulation of key gene expression or via epigenetic mechanisms such as DNA methylation.

### 2.6. Peptide Hormones in Milk/Leptin

Human milk contains a variety of endogenously synthesized peptide hormones and cytokines, which are not present or not active in infant formula. These include leptin, adiponectin, ghrelin, insulin, insulin-like growth factor-1, resistin, nesfatin-1, copeptin, and apelin, among others, which play a role in the regulation of energy metabolism and body weight [198,199]. Hence, it is reasonable to anticipate that these hormones, whose levels in breast milk are variable depending on maternal conditions, possess the potential to influence newborn growth and metabolism, and metabolic health in the long-term. However, with exceptions, the precise functions of most of these compounds in milk and their potential effects on metabolic programming are largely unknown [198,199,200]. Leptin, in particular, emerges as one of the most biologically relevant, with evidence highlighting its crucial role in the early metabolic programming of newborns [201,202]. There is also some limited evidence of the potential impact of other hormones like insulin, adiponectin, and ghrelin in milk on infant growth and development, but their precise effects remain uncertain [9,199]. Here, we will thus focus on leptin ingested during lactation as a metabolic programming factor.

#### Leptin

Leptin is a pleiotropic hormone mainly produced and secreted by the AT in proportion to the size of fat reserves. It plays a crucial role at the central level in regulating energy homeostasis by suppressing appetite and increasing energy expenditure [203,204]. Beyond its central action, leptin also plays a significant role in the AT, exerting different effects depending on the depot and the type of adipocytes. These effects encompass regulating lipid metabolism, adipogenesis, thermogenesis, browning, apoptosis, and inflammation [205]. 

Leptin is also produced by the mammary epithelium [206] and is present in breast milk [207,208], but not in infant formula [209]. Breast milk leptin seems essential for neonate development and has been proposed as one of the main factors that contribute to the benefits of breastfeeding in comparison to infant formula feeding [202]. We unveiled a novel function of leptin as a crucial nutrient during lactation. Through animal studies, we demonstrated a direct cause–effect relationship, underscoring the significance of leptin intake in the early postnatal period for the metabolic programming of neonates [201,210]. We also obtained relevant evidence from human studies that suggest milk-borne leptin protects infants from excess weight gain [211]. Leptin ingested during lactation exerts both short- and long-term regulatory actions at the central and peripheral levels, influencing metabolic programming on the neonate and shaping the development and function of critical tissues and organs, such as the hypothalamus and AT, which are pivotal in maintaining energy balance [205]. Hence, leptin’s role during this critical developmental period carries significant implications for regulating body weight and influencing the risk of obesity and related comorbidities.

Interestingly, the amount of leptin ingested in breast milk affects infant weight gain. Several independent studies have found a negative association between breast milk leptin levels and infant weight gain during lactation [211,212,213,214,215]. This provides evidence in humans of the significant role of leptin intake during this critical development period. Leptin levels in breast milk vary depending on maternal adiposity, and a positive correlation has been consistently described between maternal body mass index (BMI) or adiposity and the concentration of leptin in breast milk [202,211]. However, some of the human studies have not found such a correlation between leptin in milk and anthropometric parameters of infants [216,217,218,219]. These disparities have been attributed, at least in part, to bias arising from the inclusion of women with obesity. Maternal obesity appears to interfere with leptin action through mechanisms not yet clarified [202].

Animal studies have provided direct evidence of the critical role of leptin ingested during lactation. Male neonate rats supplemented with physiological doses of leptin during lactation were more resistant to the development of overweight/obesity [201,220,221]. Concretely, the leptin-supplemented animals showed in adulthood lower body weight and a lower adiposity index than untreated animals, both under a normal fat and a high-fat diet [201,220,221]. Moreover, they also exhibited improved insulin sensitivity and were protected from the development of obesity-related metabolic complications in adulthood, such as liver steatosis, when exposed to the dietary stressor of a high-fat diet [201,220,221].

The beneficial outcomes of leptin intake during lactation have primarily been linked to a greater imprinted leptin sensitivity at both central and peripheral levels, along with a reduced propensity for age- and diet-induced leptin resistance [202]. This has been related to changes in the expression of genes pivotal to the leptin action in central and peripheral tissues [201,221]. Specifically, in the AT, neonatal leptin supplementation has been shown to sustain the abundance of leptin receptors among animals on a high-fat diet. In contrast, non-supplemented animals exhibited a decline in these receptors in the mesenteric and retroperitoneal depots. Besides sustaining AT leptin sensitivity, neonatal leptin supplementation is associated with an enhanced fatty acid oxidative capacity in AT and a better ability to handle excess fuel [221].

Research conducted in animal models has provided valuable insights into the specific biological functions of leptin during lactation and the underlying mechanisms. During this period, rather than playing a main role in energy balance, leptin plays a crucial neurotrophic role, significantly contributing to the proper development of hypothalamic circuits involved in the regulation of body weight [202,222,223]. In rodents, this action is temporally restricted to the second postnatal week, coinciding with a transient surge in circulating leptin levels [222]. Disruptions in this leptin peak due to factors such as gestational calorie restriction [224], or the lack of leptin during this critical window, as observed in leptin-deficient rodents [222], can adversely affect the neuronal organization of hypothalamic nuclei and lead to impaired energy homeostasis regulation in adulthood. In animal models, it has been shown that treatment with exogenous leptin during the lactation period, but not in adulthood, promotes neuronal development and allows the recovery of neuronal projections of the arcuate nucleus interrupted in genetically leptin-deficient mice [222]. Likewise, oral supplementation with physiological doses of leptin during lactation reverses much of the alterations in the CNS caused by moderate gestational calorie restriction [225,226]. This was translated into a healthier phenotype in adulthood [227]. Specifically, increased fat accumulation and other alterations related to the metabolic syndrome, such as hepatic fat accumulation, hypertriglyceridemia, and insulin resistance, which are particularly evident when exposed to the stress of a diet rich in fats and sugars (Western diet) in adulthood, were largely prevented [227].

The molecular mechanisms of the effects of leptin on metabolic programming have only been sparsely studied, but its potential connection to *POMC* gene methylation seems of relevance. The evidence from animal models indicates a complex relationship between pre- and postnatal exposures that impact epigenetic mechanisms linked to the *POMC* gene [228]. Considering the established connection between leptin and the upregulation of POMC in the hypothalamus, we explored in rats whether leptin supplementation during the suckling period could impact the programming of hypothalamic neuropeptides, including POMC [229]. We found that the reduced body weight and food intake that exhibited leptin-treated animals in adulthood was accompanied by significantly increased methylation at a CpG site within the promoter of the *Pomc* gene when animals were fed a normal diet, while methylation levels were lower when fed a high-fat diet compared to vehicle-treated animals [229].

In addition to the effects of leptin on the development of CNS structures, leptin can exert programming effects on the development and function of the AT [230]. Evidence has been obtained from animal models exposed to adverse conditions during pregnancy. For example, gestational malnutrition in rodents affects the development of peripheral nervous system structures, including the sympathetic innervation of inguinal WAT [231] and BAT [232]. This alteration might relate to the observed lack of leptin surge during the second postnatal week in the offspring born to calorie-restricted dams [224]. The SNS influences the size of AT: the sympathetic denervation of AT decreases the content of norepinephrine and increases fat mass and the number of fat cells [233]. Accordingly, the reduced sympathetic innervation of the inguinal fat depot was associated with increased fat accumulation in adulthood [231]. In turn, in BAT, the reduced innervation may account for the diminished thermogenic capacity and the greater sensitivity to cold (with a higher body temperature decline when exposed to it) observed in these animals at a juvenile age [232]. Interestingly, leptin supplementation at physiological doses throughout lactation restored the sympathetic innervation and normalized the expression of genes related to lipolysis and fatty acid oxidation in the inguinal AT depot [234], and prevented the programmed predisposition for increased fat accumulation and other metabolic alterations in adulthood caused by gestational malnutrition [227].

It is known that circulating leptin participates in both the induction of BAT thermogenesis and WAT browning [205]. Concretely, in BAT, leptin activates thermogenesis and increases the production of UCP1 [235]. It also induces browning in WAT, resulting in the appearance of beige adipocytes expressing UCP1 and other browning markers [205]. These effects are primarily mediated by interactions with leptin-responsive neurons in the CNS, which govern the outflow of the SNS to both BAT and WAT [236,237]. Leptin may also exert direct (autocrine/paracrine) effects on these tissues, further influencing their metabolic activities [238], although the direct effects of leptin on these processes are probably more limited and remain incompletely understood. In any case, the role of endogenous leptin in stimulating energy expenditure is clear. However, the potential impact of leptin ingested during lactation on thermogenesis and browning processes remains largely unexplored. In a study in which newborn rats were supplemented with leptin during lactation, no significant effects on BAT weight or UCP1 mRNA and protein levels in adulthood were found [201]. Consequently, differences in BAT thermogenesis do not seem to significantly contribute to the observed effects of leptin during lactation on later body weight, namely resistance to the development of overweight/obesity. However, considering the influence of leptin during lactation on the development of peripheral nervous system structures, it is plausible that these animals might exhibit an enhanced response to thermogenic stimuli. The long-term effect of leptin on other hormones has not been directly explored, but leptin supplementation during suckling in the offspring of gestational calorie-restricted rats has been reported to normalize the decreased plasma T3 levels and altered T3 signaling in WAT [234]. This is of note, given the role of thyroid hormones in the regulation of lipogenesis and lipolysis in WAT and thermogenesis in BAT [239,240].

The effects of leptin on metabolic programming during lactation may be influenced by maternal metabolic status and diet during the perinatal period. Specifically, in animal models, the effects of leptin supplementation during the suckling period are less evident in the offspring of mothers fed a Western diet than in the offspring of mothers fed a standard diet, regardless of body weight [197]. These results could have additional significance in humans and partly explain the lack of a clear inverse association between leptin levels in breast milk and infant body weight in women with overweight/obesity [202]. It should also be mentioned that, in contrast to the aforementioned beneficial effects of orally administered leptin at physiological doses during the suckling period, leptin injected at pharmacologic doses in neonatal rats may lead to adult obesity [241]. However, the injection into neonatal rats born to malnourished mothers did normalize the altered phenotype in adulthood, including body weight, fat mass, calorie intake, locomotor activity, as well as circulating leptin and insulin [241,242].

Related to the abovementioned aspects, it should be noted that the effects of leptin during lactation may be influenced by other adipokines, such as adiponectin. The relationship between leptin and adiponectin, often represented as the leptin to adiponectin (L/A) ratio, plays a significant role in metabolic conditions, such as insulin resistance, and has been suggested as a marker of AT dysfunction and inflammation [243,244]. However, the potential impact of the L/A ratio in milk on the long-term metabolic health of infants has been barely explored. In rats, the milk L/A ratio at day 15 of lactation positively correlated with the body fat mass of female offspring in adulthood, whereas absolute levels of both hormones per se did not show significant correlations [163]. A similar trend was observed in males. These findings suggest that the milk L/A ratio might play a role in the future metabolism and health of newborns [163], but further studies are needed to confirm this association.

Finally, although our focus has primarily been on the effects of leptin as a component of milk during lactation, we note that leptin may also play a role during gestation in programming the structure and function of AT, as well as other key organs and tissues [205]. This may have implications for body weight control and the risk of obesity. This is particularly relevant in humans because AT depots begin to develop during the second trimester of gestation, unlike rodents, where AT development occurs later, within the first two weeks after birth [30]. Therefore, in humans, the influence of hormonal and other environmental signals on AT development during gestation could be crucial in metabolic programming. Consequently, the dysregulation of leptin production and/or function during fetal development, e.g., associated with maternal obesity, may result in altered growth [245]. Interestingly, in a rat model, we have described the appearance of placenta-derived leptin in amniotic fluid by the end of gestation. Amniotic fluid leptin might be functionally important for the near-term fetus, as it was internalized into the immature stomach after being swallowed, and then transferred to fetal circulation [246]. At this time near parturition, leptin may be important to induce satiety and play a role in metabolic programming in the developing offspring. Consequently, nutritional and other environmental factors that modulate these leptin levels could be crucial for later health-related outcomes and energy metabolism.

### 2.7. Myo-Inositol

Myo-inositol (MI), the primary isomeric form of inositol, is a carbocyclic sugar with six hydroxyl groups that can be acquired from the diet or endogenously synthesized from glucose-6-phosphate by myo-inositol-3-phosphate synthase. It plays a crucial role in various biological processes within mammalian physiology. Specifically, MI is the precursor for phosphatidylinositol (PI)-4,5-bisphosphate. This compound acts as the substrate for a particular phospholipase C enzyme, leading to the production of inositol 1,4,5-trisphosphate and diacylglycerol, which function as second messengers in cellular signaling pathways [247].

MI has been proposed as an insulin-sensitizing substance in treating metabolic syndrome [248,249]. Animal studies have reported that oral MI treatment improves glucose tolerance and insulin sensitivity, as well as reduces WAT accretion in monkeys [250] and mice [251,252]. In a study in postmenopausal women with metabolic syndrome, MI supplementation led to better glucose levels, insulin sensitivity, lipid profile, and blood pressure compared to the placebo [253]. Notably, 20% of women in the MI group no longer met the criteria for metabolic syndrome after the supplementation, a significant improvement over the control group. In pregnant women with overweight and obesity, supplementing with MI during pregnancy reduced the incidence of gestational diabetes and its associated complications, including premature birth and gestational hypertension [254,255]. However, the quality of the evidence is still low, suggesting the need for further studies to corroborate these latter findings [254,255].

It has been hypothesized that MI could play a significant role in neonatal development as a component of breast milk [256]. Of note, breast milk generally contains a higher concentration of MI compared to infant formula [257,258], and hence breastfed infants exhibit greater serum levels compared to formula-fed infants [257,258,259]. Interestingly, MI levels are increased in milk from rats exposed to moderate calorie restriction during lactation [256], which is a model associated with a better protection of the offspring against obesity and insulin resistance in later life [260,261]. However, studies addressing the effects of MI present in milk are scarce. We recently investigated the impact of MI supplementation at physiological doses during the suckling period both in control rats and the offspring of rats exposed to gestational calorie restriction [198,226,262]. The effects were more evident in the offspring of dams undergoing gestational calorie restriction. In these animals, MI supplementation demonstrated efficacy in improving metabolic health and ameliorating insulin resistance and hypertriglyceridemia programmed by maternal undernutrition, which was particularly pronounced when combined with exposure to an obesogenic diet in adulthood [198]. The effects were more apparent in males, who were also more sensitive than females to exposure to the obesogenic diet. Moreover, MI supplementation also partially reversed the postnatal phenotypic consequences linked to fetal malnutrition in terms of hypothalamic development, leading to an increased number of neurons in the ARC and PVN nuclei, albeit in a sex-dependent manner [226]. Interestingly, some of the effects of MI supplementation observed in these preclinical studies were comparable, to some extent, to those observed for leptin, both facilitating the reversal of adverse outcomes resulting from gestational undernutrition. Therefore, a potential role of MI empowering the action of leptin naturally ingested with milk has been suggested [226].

Furthermore, MI ingested during the suckling period impacted the development of the AT, favoring the browning process and enhancing thermogenesis capacity, thus having beneficial effects on metabolism and energy expenditure [262]. Specifically, at the age of 7 months and after two months of exposure to the stress of a Western diet, animals that were supplemented with MI during suckling exhibited elevated expression levels of *Ucp1*, *Hoxc9*, and *Cidea* in WAT, with variations based on the depot and sex of the animals [262]. Again, the effects were most pronounced in offspring born to dams undergoing gestational calorie restriction. Nevertheless, some effects were also appreciated in the progeny of control rats since they exhibited a greater expression of *Hoxc9* in the inguinal WAT depot (in males and females) and *Ucp1* in the retroperitoneal WAT (only in males). In BAT, MI supplementation during suckling allowed the normalization of UCP1 protein levels, which were significantly diminished in the female offspring of rats exposed to gestational calorie restriction [262].

The mechanisms underlying the metabolic programming effects of MI ingested during the suckling period remain incompletely understood. One potential avenue is the ability of MI to favor leptin action during this critical development stage of lactation. This is supported by the partially comparable effects observed with MI and leptin supplementation in neonatal rats, particularly on hypothalamic development [226]. Moreover, a possible role of the brain-derived neurotrophic factor (BDNF) in mediating the effects of MI during the suckling period has been proposed [262]. BDNF is a multifaceted neurotrophin that regulates nerve growth and influences energy balance by modulating thermogenesis in BAT, promoting WAT browning, and enhancing insulin sensitivity and glucose uptake in skeletal muscle and AT [263]. Interestingly, the offspring of rats supplemented with MI during suckling displayed elevated circulating BDNF levels, especially those born to dams undergoing gestational calorie restriction [262]. Therefore, the activation of BDNF signaling could partially contribute to the observed positive effects of MI supplementation during the early postnatal period. The potential impact of MI on leptin sensitization during lactation, referenced above, is also worth considering, although additional confirmation is needed.

## 3. Concluding Remarks and Future Directions

The rising prevalence of obesity in humans has prompted considerable research into the relationship between AT and early life conditions. It is widely recognized that nutrition during the critical window of plasticity, mainly represented by the first 1000 days, plays a pivotal role in mammalian early growth and development, exerting long-lasting effects on metabolic health. AT plays a crucial role in regulating energy homeostasis and metabolism, and BAT function and WAT browning are promising therapeutic targets for combating weight gain. It is clear that cellular and metabolic aspects of AT biology—e.g., cellularity, sympathetic innervation, and adipose progenitor cells’ features/capacity for browning—are programmable by specific nutritional compounds acting in critical developmental periods.

**Figure 2:**
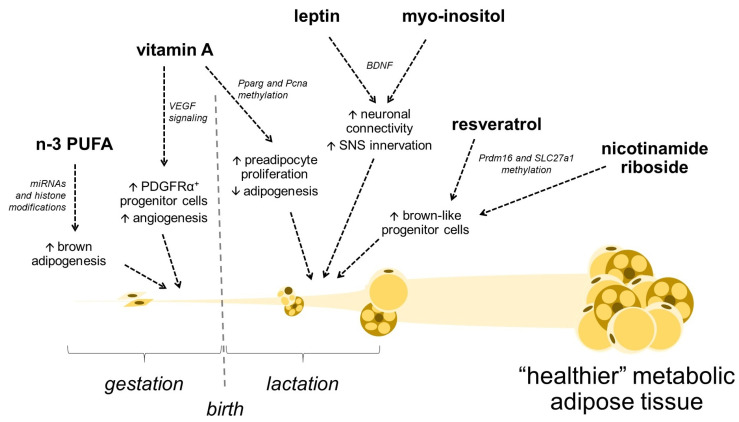
Adipose tissue features impacted by the indicated nutritional interventions during gestation and/or lactation and postulated mechanisms.

Research in animal models is essential to the field of metabolic programming because it allows the precise control of the timing and nature of the exposures and delves into molecular and mechanistic aspects. Initial studies in the field focused on the adverse programming effects of maternal over- and undernutrition and unbalanced dietary macronutrient composition. Regarding the micronutrients, the initial focus was on the micronutrients involved in the 1 C metabolism pathway—owing to the importance of this pathway for epigenetic reactions that may be at the root of long-lasting biological effects—and the impact of micronutrient deficiencies. More recently, interest shifted to specific dietary compounds that may exert positive programming effects or help counteract malprogramming caused by adverse in utero exposures. Here, we adopted an adipocentric view and reviewed nutritional compounds for which beneficial programming activity is documented involving the modulation of specific aspects of AT biology. The different aspects affected and postulated mechanisms are schematized in Figure 2, and Table 1 summarizes key studies reviewed documenting specifically the enhancement of fat browning by early exposure to nutrients/food bioactive compounds. Nutritional compounds with beneficial programming activity but that have not been studied so far for their specific impact on AT pathways beyond changes in body fat content are not included in this review, though notable examples exist. This is the case of taurine, a semi-essential amino acid that is added to milk formula for babies and to parental solution for premature babies, and whose supplementation to mothers has been shown to counteract adverse programming associated with gestational protein restriction [264], fructose feeding [265], and cafeteria diet feeding [266] in animal studies.

Studies reviewed herein—carried out in various animal models, with different interventions during pregnancy and/or lactation, and targeting mothers or offspring—have shed light on the effects of specific compounds on the development and function of AT, its long-term consequences on health status, and the underlying mechanisms (Figure 1 and Figure 2). Although here we focus on AT, and partly on the hypothalamus, as the main organs involved in energy homeostasis, we must consider that programming generally encompasses various tissues and organs, therefore affecting the entire physiology of the organism. In this sense, interventions in mothers during pregnancy and/or lactation with specific compounds, such as nicotinamide riboside, vitamin A, resveratrol, and PUFA, have shown effects on the development of AT and have been related to improved functionality in adulthood. On the other hand, changes in the composition of certain miRNAs (e.g., miR-222) in milk due to dietary interventions (e.g., an obesogenic diet) and/or an altered metabolic state (obesity and/or gestational diabetes) have been related to alterations in the development of the AT and result in greater adiposity in adulthood. Strategies to ensure an optimal maternal intake of the aforementioned compounds and to effectively control the levels of specific miRNAs in milk are thus of potential interest for the metabolic programming of a healthy adult phenotype.

Regarding lactation, human milk stands as the gold standard for infant nutrition, offering a rich array of nutrients, bioactive compounds, and stimuli essential for infant growth and development. Moreover, breast milk plays a crucial role in metabolic programming. A range of nutritional components naturally present in breast milk, such as peptide hormones (leptin), vitamins (vitamin A, nicotinamide riboside), myo-inositol, and resveratrol, have been shown to possess protective effects against obesity susceptibility in animal studies involving direct interventions in pups during the suckling period. These compounds may affect the development of neuroendocrine circuits at the central and/or peripheral level (as suggested for leptin and myo-inositol) or AT development and function through local epigenetic modifications (as suggested by vitamin A, resveratrol and nicotinamide riboside) and effects on progenitor cells, therefore influencing AT cellularity and metabolic capacities. These effects may ultimately impact, at least in part, the ability for AT remodeling in response to biological signals and/or conditions, among other processes. It is crucial to emphasize that these bioactive compounds may be present in lower amounts (e.g., myo-inositol, provitamin A carotenoids, polyphenols) or even absent or inactive (e.g., leptin) in infant formula compared to breast milk. These differences may contribute to the long-term health advantages of breast milk over infant formula, as appears particularly evident for leptin. Furthermore, the levels of these compounds in breast milk can be affected by maternal factors, such as diet or metabolic health status (e.g., obesity or gestational diabetes), potentially influencing offspring growth and development.

The metabolic programming activity of early life nutritional exposures interacts with diet later in life to determine metabolic health in adulthood. The programming “legacy” can be masked or, on the contrary, unveiled/reinforced depending on the overall characteristics of the contemporary diet. However, earlier preventive interventions are considered more effective in reducing the risk of non-communicable metabolic diseases and preventing their transgenerational conditioning than interventions in affected adults [267]. The earlier interventions may favor life-long improved functional capacity, better responses to dietary challenges, and enhanced responsiveness to beneficial food compounds or conditions. For instance, early life interventions impinging on the population of progenitor cells in WAT capable of brown/beige adipogenesis can remain influential for WAT browning in older animals, or even reveal themselves as such only in older animals, after rounds of progenitor cell proliferation and de novo adipogenesis linked to aging and eventual obesogenic diet feeding (as in the case of nicotinamide riboside and resveratrol supplementation during suckling [43,44]).

Optimizing maternal nutrition during gestation and lactation holds promise as a feasible and effective strategy for combating obesity and associated metabolic disorders in offspring. However, our understanding of the specific roles of many bioactive compounds present in breast milk remains limited. Long-term human studies are recommended, and intervention studies in animal models may help gain insights into the molecular mechanisms through which micronutrients and bioactive compounds influence AT remodeling and browning capacity, thereby opening up novel avenues for targeted interventions, with broad implications for public health. In particular, dietary guidelines for optimal nutritional interventions could be updated to mitigate the risk of obesity and metabolic disease across generations. However, further research is imperative to translate these findings appropriately and effectively into clinical practice.

## Figures and Tables

**Table 1:** Key studies reviewed regarding early exposure to nutrients and adipose tissue browning.

Nutrient/Bioactive Compound	Species	Intervention in	Dose	Offspring Studied at/in	Offspring Sex Studied	Main Intervention Outcomes in the Offspring	Reference
Nicotinamide riboside (NR)	NMRI mice	pups during lactation	from 24 μg per pup on PND-2 to 45 μg on PND-20 (~15-fold the total B3 quantity derived from maternal milk)	adulthood after a 10-week NFD/HFD challenge starting at age 3 months	male and female	Improved responses to the HFD and increased signs of scWAT browning and BAT activation after the HFD in the male mice	[43]
Nicotinamide riboside (NR)	NMRI mice	pups during lactation	from 24 μg per pup on PND-2 to 45 μg on PND-20 (~15-fold the total B3 quantity derived from maternal milk)	PND-35	male and female	Increased spontaneous beige adipogenesis in primary scWAT cultures from NR-supplemented male mice	[44]
Nicotinamide riboside (NR)	NMRI mice	pups during lactation	from 24 μg per pup on PND-2 to 45 μg on PND-20 (~15-fold the total B3 quantity derived from maternal milk)	PND-35 and adulthood after a 10-week NFD/HFD challenge starting at age 3 months	male	Decreased *Dnmt3b* expression in scWAT at PND-35; changes in methylation status of browning-related genes and its HFD-induced dynamics in the adult scWAT	[45]
Vitamin A (VA)	C57BL/6 mice	mothers during gestation and lactation	2-fold excess VA as retinyl acetate in drinking water	weaning (PND-21) and adulthood after 5 months of HFD starting at PND-30	male	Increased beige/brown adipogenesis in WAT and BAT at weaning; protection against HFD-induced obesity in adulthood	[86]
Resveratrol (RES)	C57BL/6 mice	mothers during gestation and lactation, as part of a control diet and a HFD	0.2% RES in the diets, equivalent to ~200 mg per kg bw per day	weaning (PND-21) and adulthood after 11 weeks of HFD starting at weaning	male	Increased beige/brown adipogenesis in WAT and BAT in offspring of HFD-fed mothers; protection against HFD-induced obesity in adulthood	[110]
Resveratrol (RES)	NMRI mice	pups during lactation	2 mg per kg bw per day from PND-2 to PND-20	adulthood after a 10-week NFD/HFD challenge starting at age 3 months	male and female	Delayed HFD-induced bw gain in the RES male mice; increased signs of scWAT browning after both NFD and HFD in the male mice	[43]
Resveratrol (RES)	NMRI mice	pups during lactation	2 mg per kg bw per day from PND-2 to PND-20	PND-35	male and female	Increased beige and brown adipogenesis in primary cultures derived from scWAT and BAT of RES-supplemented males	[44]
Resveratrol (RES)	NMRI mice	pups during lactation	2 mg per kg bw per day from PND-2 to PND-20	PND-35 and adulthood after a 10-week NFD/HFD challenge starting at age 3 months	male	Decreased *Dnmt3a* and *Dnmt3b* gene expression in scWAT at PND-35; changes in the methylation status of browning-related genes and its HFD-induced dynamics in the adult scWAT	[45]
n-3 polyunsaturated fatty acids (PUFA)	C57BL/6 mice	mothers during gestation and lactation	3% PUFA from fish oil in the diet	weaning (PND-21) and age 11 weeks	males and females	Enhanced BAT development at weaning; epigenetic changes in BAT at weaning; increased total energy expenditure and cold-resistance in adulthood (age 11 weeks); higher BAT activity and scWAT browning after cold-exposure in adulthood	[144]
Myo-inositol (MI)	Wistar rats	pups of control (CON) and 25%-calorie-restricted (CR) pregnant rats during lactation	twice the average quantity derived from maternal milk	weaning (PND-21) and at 7 months of age, after a 2-month Western diet challenge	males and females	upregulation of circulating BDNF levels at weaning and of browning markers in WAT and BAT in the adult animals, especially those born to CR mothers	[262]

Abbreviations: BAT, brown adipose tissue; *Dnmt*, DNA methyltransferase gene; HFD, high-fat diet; PND, postnatal day; scWAT, subcutaneous white adipose tissue.

## References

[1] WHO Obesity and Overweight. Fact Sheet. http://www.who.int/mediacentre/factsheets/fs311/en/.

[2] Wearing S.C., Hennig E.M., Byrne N.M., Steele J.R., Hills A.P. (2006). Musculoskeletal disorders associated with obesity: A biomechanical perspective. Obes. Rev..

[3] Guh D.P., Zhang W., Bansback N., Amarsi Z., Birmingham C.L., Anis A.H. (2009). The incidence of co-morbidities related to obesity and overweight: A systematic review and meta-analysis. BMC Public Health.

[4] Pati S., Irfan W., Jameel A., Ahmed S., Shahid R.K. (2023). Obesity and Cancer: A Current Overview of Epidemiology, Pathogenesis, Outcomes, and Management. Cancers.

[5] Melson E., Ashraf U., Papamargaritis D., Davies M.J. (2024). What is the pipeline for future medications for obesity?. Int. J. Obes..

[6] Noria S.F., Shelby R.D., Atkins K.D., Nguyen N.T., Gadde K.M. (2023). Weight Regain After Bariatric Surgery: Scope of the Problem, Causes, Prevention, and Treatment. Curr. Diabetes Rep..

[7] Wilding J.P.H., Batterham R.L., Davies M., Van Gaal L.F., Kandler K., Konakli K., Lingvay I., McGowan B.M., Oral T.K., Rosenstock J. (2022). Weight regain and cardiometabolic effects after withdrawal of semaglutide: The STEP 1 trial extension. Diabetes Obes. Metab..

[8] Fall C.H.D., Kumaran K. (2019). Metabolic programming in early life in humans. Philos. Trans. R. Soc. Lond. Ser. B Biol. Sci..

[9] Pico C., Reis F., Egas C., Mathias P., Matafome P. (2021). Lactation as a programming window for metabolic syndrome. Eur. J. Clin. Investig..

[10] Vickers M.H. (2022). Early life nutrition and neuroendocrine programming. Neuropharmacology.

[11] Zhu Z., Cao F., Li X. (2019). Epigenetic Programming and Fetal Metabolic Programming. Front. Endocrinol..

[12] Sanli E., Kabaran S. (2019). Maternal Obesity, Maternal Overnutrition and Fetal Programming: Effects of Epigenetic Mechanisms on the Development of Metabolic Disorders. Curr. Genom..

[13] Marasco V., Smith S., Angelier F. (2022). How does early-life adversity shape telomere dynamics during adulthood? Problems and paradigms. Bioessays.

[14] Calatayud M., Koren O., Collado M.C. (2019). Maternal Microbiome and Metabolic Health Program Microbiome Development and Health of the Offspring. Trends Endocrinol. Metab..

[15] Zwick R.K., Guerrero-Juarez C.F., Horsley V., Plikus M.V. (2018). Anatomical, Physiological, and Functional Diversity of Adipose Tissue. Cell Metab..

[16] Cohen P., Kajimura S. (2021). The cellular and functional complexity of thermogenic fat. Nat. Rev. Mol. Cell Biol..

[17] Cinti S. (2005). The adipose organ. Prostaglandins Leukot. Essent. Fatty Acids.

[18] Luo L., Liu M. (2016). Adipose tissue in control of metabolism. J. Endocrinol..

[19] Lee M.J., Wu Y., Fried S.K. (2010). Adipose tissue remodeling in pathophysiology of obesity. Curr. Opin. Clin. Nutr. Metab. Care.

[20] Choe S.S., Huh J.Y., Hwang I.J., Kim J.I., Kim J.B. (2016). Adipose Tissue Remodeling: Its Role in Energy Metabolism and Metabolic Disorders. Front. Endocrinol..

[21] Palacios-Marin I., Serra D., Jimenez-Chillaron J., Herrero L., Todorcevic M. (2023). Adipose Tissue Dynamics: Cellular and Lipid Turnover in Health and Disease. Nutrients.

[22] Longo M., Zatterale F., Naderi J., Parrillo L., Formisano P., Raciti G.A., Beguinot F., Miele C. (2019). Adipose Tissue Dysfunction as Determinant of Obesity-Associated Metabolic Complications. Int. J. Mol. Sci..

[23] Ghesmati Z., Rashid M., Fayezi S., Gieseler F., Alizadeh E., Darabi M. (2024). An update on the secretory functions of brown, white, and beige adipose tissue: Towards therapeutic applications. Rev. Endocr. Metab. Disord..

[24] Shao M., Wang Q.A., Song A., Vishvanath L., Busbuso N.C., Scherer P.E., Gupta R.K. (2019). Cellular Origins of Beige Fat Cells Revisited. Diabetes.

[25] Bonet M.L., Oliver P., Palou A. (2013). Pharmacological and nutritional agents promoting browning of white adipose tissue. Biochim. Biophys. Acta.

[26] Bonet M.L., Mercader J., Palou A. (2017). A nutritional perspective on UCP1-dependent thermogenesis. Biochimie.

[27] Becher T., Palanisamy S., Kramer D.J., Eljalby M., Marx S.J., Wibmer A.G., Butler S.D., Jiang C.S., Vaughan R., Schoder H. (2021). Brown adipose tissue is associated with cardiometabolic health. Nat. Med..

[28] Lecoutre S., Breton C. (2014). The cellularity of offspring’s adipose tissue is programmed by maternal nutritional manipulations. Adipocyte.

[29] Moreno-Mendez E., Quintero-Fabian S., Fernandez-Mejia C., Lazo-de-la-Vega-Monroy M.L. (2020). Early-life programming of adipose tissue. Nutr. Res. Rev..

[30] Rodgers A., Sferruzzi-Perri A.N. (2021). Developmental programming of offspring adipose tissue biology and obesity risk. Int. J. Obes..

[31] Liang X., Yang Q., Zhang L., Maricelli J.W., Rodgers B.D., Zhu M.J., Du M. (2016). Maternal high-fat diet during lactation impairs thermogenic function of brown adipose tissue in offspring mice. Sci. Rep..

[32] Pomar C.A., Pico C., Palou A., Sanchez J. (2022). Maternal Consumption of a Cafeteria Diet during Lactation Leads to Altered Diet-Induced Thermogenesis in Descendants after Exposure to a Western Diet in Adulthood. Nutrients.

[33] Savva C., Helguero L.A., Gonzalez-Granillo M., Melo T., Couto D., Buyandelger B., Gustafsson S., Liu J., Domingues M.R., Li X. (2022). Maternal high-fat diet programs white and brown adipose tissue lipidome and transcriptome in offspring in a sex- and tissue-dependent manner in mice. Int. J. Obes..

[34] Bieganowski P., Brenner C. (2004). Discoveries of nicotinamide riboside as a nutrient and conserved NRK genes establish a Preiss-Handler independent route to NAD+ in fungi and humans. Cell.

[35] Trammell S.A., Yu L., Redpath P., Migaud M.E., Brenner C. (2016). Nicotinamide Riboside Is a Major NAD+ Precursor Vitamin in Cow Milk. J. Nutr..

[36] Ummarino S., Mozzon M., Zamporlini F., Amici A., Mazzola F., Orsomando G., Ruggieri S., Raffaelli N. (2017). Simultaneous quantitation of nicotinamide riboside, nicotinamide mononucleotide and nicotinamide adenine dinucleotide in milk by a novel enzyme-coupled assay. Food Chem..

[37] Canto C., Houtkooper R.H., Pirinen E., Youn D.Y., Oosterveer M.H., Cen Y., Fernandez-Marcos P.J., Yamamoto H., Andreux P.A., Cettour-Rose P. (2012). The NAD(+) precursor nicotinamide riboside enhances oxidative metabolism and protects against high-fat diet-induced obesity. Cell Metab..

[38] Mehmel M., Jovanovic N., Spitz U. (2020). Nicotinamide Riboside-The Current State of Research and Therapeutic Uses. Nutrients.

[39] Remie C.M.E., Roumans K.H.M., Moonen M.P.B., Connell N.J., Havekes B., Mevenkamp J., Lindeboom L., de Wit V.H.W., van de Weijer T., Aarts S. (2020). Nicotinamide riboside supplementation alters body composition and skeletal muscle acetylcarnitine concentrations in healthy obese humans. Am. J. Clin. Nutr..

[40] Trammell S.A., Weidemann B.J., Chadda A., Yorek M.S., Holmes A., Coppey L.J., Obrosov A., Kardon R.H., Yorek M.A., Brenner C. (2016). Nicotinamide Riboside Opposes Type 2 Diabetes and Neuropathy in Mice. Sci. Rep..

[41] Canto C., Gerhart-Hines Z., Feige J.N., Lagouge M., Noriega L., Milne J.C., Elliott P.J., Puigserver P., Auwerx J. (2009). AMPK regulates energy expenditure by modulating NAD+ metabolism and SIRT1 activity. Nature.

[42] Ear P.H., Chadda A., Gumusoglu S.B., Schmidt M.S., Vogeler S., Malicoat J., Kadel J., Moore M.M., Migaud M.E., Stevens H.E. (2019). Maternal Nicotinamide Riboside Enhances Postpartum Weight Loss, Juvenile Offspring Development, and Neurogenesis of Adult Offspring. Cell Rep..

[43] Serrano A., Asnani-Kishnani M., Rodriguez A.M., Palou A., Ribot J., Bonet M.L. (2018). Programming of the Beige Phenotype in White Adipose Tissue of Adult Mice by Mild Resveratrol and Nicotinamide Riboside Supplementations in Early Postnatal Life. Mol. Nutr. Food Res..

[44] Asnani-Kishnani M., Rodriguez A.M., Serrano A., Palou A., Bonet M.L., Ribot J. (2019). Neonatal Resveratrol and Nicotinamide Riboside Supplementations Sex-Dependently Affect Beige Transcriptional Programming of Preadipocytes in Mouse Adipose Tissue. Front. Physiol..

[45] Serrano A., Asnani-Kishnani M., Couturier C., Astier J., Palou A., Landrier J.F., Ribot J., Bonet M.L. (2020). DNA Methylation Changes are Associated with the Programming of White Adipose Tissue Browning Features by Resveratrol and Nicotinamide Riboside Neonatal Supplementations in Mice. Nutrients.

[46] Serrano A., Palou A., Bonet M.L., Ribot J. (2022). Nicotinamide Riboside Supplementation to Suckling Male Mice Improves Lipid and Energy Metabolism in Skeletal Muscle and Liver in Adulthood. Nutrients.

[47] Cao L., Choi E.Y., Liu X., Martin A., Wang C., Xu X., During M.J. (2011). White to brown fat phenotypic switch induced by genetic and environmental activation of a hypothalamic-adipocyte axis. Cell Metab..

[48] Kahn C.R., Wang G., Lee K.Y. (2019). Altered adipose tissue and adipocyte function in the pathogenesis of metabolic syndrome. J. Clin. Investig..

[49] Balmer J.E., Blomhoff R. (2002). Gene expression regulation by retinoic acid. J. Lipid Res..

[50] Bonet M.L., Ribot J., Felipe F., Palou A. (2003). Vitamin A and the regulation of fat reserves. Cell Mol. Life Sci..

[51] Bonet M.L., Ribot J., Palou A. (2012). Lipid metabolism in mammalian tissues and its control by retinoic acid. Biochim. Biophys. Acta.

[52] Brun P.J., Yang K.J., Lee S.A., Yuen J.J., Blaner W.S. (2013). Retinoids: Potent regulators of metabolism. Biofactors.

[53] Blaner W.S. (2019). Vitamin A signaling and homeostasis in obesity, diabetes, and metabolic disorders. Pharmacol. Ther..

[54] Felipe F., Bonet M.L., Ribot J., Palou A. (2003). Up-regulation of muscle uncoupling protein 3 gene expression in mice following high fat diet, dietary vitamin A supplementation and acute retinoic acid-treatment. Int. J. Obes. Relat. Metab. Disord..

[55] Jeyakumar S.M., Vajreswari A., Giridharan N.V. (2008). Vitamin A regulates obesity in WNIN/Ob obese rat; independent of stearoyl-CoA desaturase-1. Biochem. Biophys. Res. Commun..

[56] Amengual J., Gouranton E., van Helden Y.G., Hessel S., Ribot J., Kramer E., Kiec-Wilk B., Razny U., Lietz G., Wyss A. (2011). Beta-carotene reduces body adiposity of mice via BCMO1. PLoS ONE.

[57] Ribot J., Felipe F., Bonet M.L., Palou A. (2001). Changes of adiposity in response to vitamin A status correlate with changes of PPAR gamma 2 expression. Obes. Res..

[58] Berry D.C., Noy N. (2009). All-trans-retinoic acid represses obesity and insulin resistance by activating both peroxisome proliferation-activated receptor beta/delta and retinoic acid receptor. Mol. Cell Biol..

[59] Berry D.C., DeSantis D., Soltanian H., Croniger C.M., Noy N. (2012). Retinoic acid upregulates preadipocyte genes to block adipogenesis and suppress diet-induced obesity. Diabetes.

[60] Coronel J., Yu J., Pilli N., Kane M.A., Amengual J. (2022). The conversion of beta-carotene to vitamin A in adipocytes drives the anti-obesogenic effects of beta-carotene in mice. Mol. Metab..

[61] Alvarez R., de Andres J., Yubero P., Vinas O., Mampel T., Iglesias R., Giralt M., Villarroya F. (1995). A novel regulatory pathway of brown fat thermogenesis. Retinoic acid is a transcriptional activator of the mitochondrial uncoupling protein gene. J. Biol. Chem..

[62] Puigserver P., Vazquez F., Bonet M.L., Pico C., Palou A. (1996). In vitro and in vivo induction of brown adipocyte uncoupling protein (thermogenin) by retinoic acid. Biochem. J..

[63] Herz C.T., Kiefer F.W. (2020). The Transcriptional Role of Vitamin A and the Retinoid Axis in Brown Fat Function. Front. Endocrinol..

[64] Mercader J., Ribot J., Murano I., Felipe F., Cinti S., Bonet M.L., Palou A. (2006). Remodeling of white adipose tissue after retinoic acid administration in mice. Endocrinology.

[65] Wang B., Fu X., Liang X., Deavila J.M., Wang Z., Zhao L., Tian Q., Zhao J., Gomez N.A., Trombetta S.C. (2017). Retinoic acid induces white adipose tissue browning by increasing adipose vascularity and inducing beige adipogenesis of PDGFRalpha(+) adipose progenitors. Cell Discov..

[66] Lobo G.P., Amengual J., Li H.N., Golczak M., Bonet M.L., Palczewski K., von Lintig J. (2010). β,β-carotene decreases peroxisome proliferator receptor gamma activity and reduces lipid storage capacity of adipocytes in a β,β-carotene oxygenase 1-dependent manner. J. Biol. Chem..

[67] Amengual J., Ribot J., Bonet M.L., Palou A. (2008). Retinoic acid treatment increases lipid oxidation capacity in skeletal muscle of mice. Obesity.

[68] Amengual J., Ribot J., Bonet M.L., Palou A. (2010). Retinoic acid treatment enhances lipid oxidation and inhibits lipid biosynthesis capacities in the liver of mice. Cell Physiol. Biochem..

[69] Amengual J., Garcia-Carrizo F.J., Arreguin A., Musinovic H., Granados N., Palou A., Bonet M.L., Ribot J. (2018). Retinoic Acid Increases Fatty Acid Oxidation and Irisin Expression in Skeletal Muscle Cells and Impacts Irisin In Vivo. Cell Physiol. Biochem..

[70] Bonet M.L., Canas J.A., Ribot J., Palou A. (2015). Carotenoids and their conversion products in the control of adipocyte function, adiposity and obesity. Arch. Biochem. Biophys..

[71] Zulet M.A., Puchau B., Hermsdorff H.H., Navarro C., Martinez J.A. (2008). Vitamin A intake is inversely related with adiposity in healthy young adults. J. Nutr. Sci. Vitaminol..

[72] Canas J.A., Lochrie A., McGowan A.G., Hossain J., Schettino C., Balagopal P.B. (2017). Effects of Mixed Carotenoids on Adipokines and Abdominal Adiposity in Children: A Pilot Study. J. Clin. Endocrinol. Metab..

[73] Duester G. (2008). Retinoic acid synthesis and signaling during early organogenesis. Cell.

[74] Bastos Maia S., Rolland Souza A.S., Costa Caminha M.F., Lins da Silva S., Callou Cruz R., Carvalho Dos Santos C., Batista Filho M. (2019). Vitamin A and Pregnancy: A Narrative Review. Nutrients.

[75] WHO Safe Vitamin A Dosage during Pregnancy and Lactation: Recommendations and Report of a Consultation. World Health Organization. https://www.who.int/publications/i/item/WHO-NUT-98.4.

[76] EFSA Panel on Dietetic Products, Nutrition, and Allergies (NDA) (2015). Scientific Opinion on Dietary Reference Values for vitamin A. EFSA J..

[77] Zielinska M.A., Wesolowska A., Pawlus B., Hamulka J. (2017). Health Effects of Carotenoids during Pregnancy and Lactation. Nutrients.

[78] Sommerburg O., Meissner K., Nelle M., Lenhartz H., Leichsenring M. (2000). Carotenoid supply in breast-fed and formula-fed neonates. Eur. J. Pediatr..

[79] Chan G.M., Chan M.M., Gellermann W., Ermakov I., Ermakova M., Bhosale P., Bernstein P., Rau C. (2013). Resonance Raman spectroscopy and the preterm infant carotenoid status. J. Pediatr. Gastroenterol. Nutr..

[80] Vishwanathan R., Panagos P., Sen S. (2014). Breast milk carotenoid concentrations are decreased in obese mothers. FASEB J..

[81] Panagos P.G., Vishwanathan R., Penfield-Cyr A., Matthan N.R., Shivappa N., Wirth M.D., Hebert J.R., Sen S. (2016). Breastmilk from obese mothers has pro-inflammatory properties and decreased neuroprotective factors. J. Perinatol. Off. J. Calif. Perinat. Assoc..

[82] Kuri-Harcuch W. (1982). Differentiation of 3T3-F442A cells into adipocytes is inhibited by retinoic acid. Differentiation.

[83] Granados N., Amengual J., Ribot J., Musinovic H., Ceresi E., von Lintig J., Palou A., Bonet M.L. (2013). Vitamin A supplementation in early life affects later response to an obesogenic diet in rats. Int. J. Obes..

[84] Redonnet A., Ferrand C., Bairras C., Higueret P., Noel-Suberville C., Cassand P., Atgie C. (2008). Synergic effect of vitamin A and high-fat diet in adipose tissue development and nuclear receptor expression in young rats. Br. J. Nutr..

[85] Arana A., Mendizabal J.A., Alzon M., Soret B., Purroy A. (2008). The effect of vitamin A supplementation on postnatal adipose tissue development of lambs. J. Anim. Sci..

[86] Wang B., Fu X., Liang X., Wang Z., Yang Q., Zou T., Nie W., Zhao J., Gao P., Zhu M.J. (2017). Maternal Retinoids Increase PDGFRalpha(+) Progenitor Population and Beige Adipogenesis in Progeny by Stimulating Vascular Development. eBioMedicine.

[87] Lee Y.H., Petkova A.P., Mottillo E.P., Granneman J.G. (2012). In vivo identification of bipotential adipocyte progenitors recruited by beta3-adrenoceptor activation and high-fat feeding. Cell Metab..

[88] Billon N., Dani C. (2012). Developmental origins of the adipocyte lineage: New insights from genetics and genomics studies. Stem Cell Rev. Rep..

[89] Wang Q.A., Tao C., Gupta R.K., Scherer P.E. (2013). Tracking adipogenesis during white adipose tissue development, expansion and regeneration. Nat. Med..

[90] Tan L., Zhang Y., Crowe-White K.M., Senkus K.E., Erwin M.E., Wang H. (2020). Vitamin A Supplementation during Suckling and Postweaning Periods Attenuates the Adverse Metabolic Effects of Maternal High-Fat Diet Consumption in Sprague-Dawley Rats. Curr. Dev. Nutr..

[91] Tan L., Zhang Y., Wang H., Haberer H. (2022). Retinoic acid promotes tissue vitamin A status and modulates adipose tissue metabolism of neonatal rats exposed to maternal high-fat diet-induced obesity. J. Nutr. Sci..

[92] Spiegler E., Kim Y.K., Wassef L., Shete V., Quadro L. (2012). Maternal-fetal transfer and metabolism of vitamin A and its precursor beta-carotene in the developing tissues. Biochim. Biophys. Acta.

[93] Musinovic H., Bonet M.L., Granados N., Amengual J., von Lintig J., Ribot J., Palou A. (2014). beta-Carotene during the suckling period is absorbed intact and induces retinoic acid dependent responses similar to preformed vitamin A in intestine and liver, but not adipose tissue of young rats. Mol. Nutr. Food Res..

[94] Arreguin A., Ribot J., Musinovic H., von Lintig J., Palou A., Bonet M.L. (2018). Dietary vitamin A impacts DNA methylation patterns of adipogenesis-related genes in suckling rats. Arch. Biochem. Biophys..

[95] Guo J., Li B., Zuo Z., Chen M., Wang C. (2019). Maternal Supplementation with beta-Carotene During Pregnancy Disturbs Lipid Metabolism and Glucose Homoeostasis in F1 Female Mice. Mol. Nutr. Food Res..

[96] Fortunato I.M., Dos Santos T.W., Ferraz L.F.C., Santos J.C., Ribeiro M.L. (2021). Effect of Polyphenols Intake on Obesity-Induced Maternal Programming. Nutrients.

[97] Silva L., Pinheiro-Castro N., Novaes G.M., Pascoal G.F.L., Ong T.P. (2019). Bioactive food compounds, epigenetics and chronic disease prevention: Focus on early-life interventions with polyphenols. Food Res. Int..

[98] Ros P., Argente J., Chowen J.A. (2021). Effects of Maternal Resveratrol Intake on the Metabolic Health of the Offspring. Int. J. Mol. Sci..

[99] Tain Y.L., Hsu C.N. (2018). Developmental Programming of the Metabolic Syndrome: Can We Reprogram with Resveratrol?. Int. J. Mol. Sci..

[100] Pan M.H., Lai C.S., Wu J.C., Ho C.T. (2013). Epigenetic and disease targets by polyphenols. Curr. Pharm. Des..

[101] Lagouge M., Argmann C., Gerhart-Hines Z., Meziane H., Lerin C., Daussin F., Messadeq N., Milne J., Lambert P., Elliott P. (2006). Resveratrol improves mitochondrial function and protects against metabolic disease by activating SIRT1 and PGC-1alpha. Cell.

[102] Baur J.A., Pearson K.J., Price N.L., Jamieson H.A., Lerin C., Kalra A., Prabhu V.V., Allard J.S., Lopez-Lluch G., Lewis K. (2006). Resveratrol improves health and survival of mice on a high-calorie diet. Nature.

[103] Wang S., Liang X., Yang Q., Fu X., Zhu M., Rodgers B.D., Jiang Q., Dodson M.V., Du M. (2017). Resveratrol enhances brown adipocyte formation and function by activating AMP-activated protein kinase (AMPK) alpha1 in mice fed high-fat diet. Mol. Nutr. Food Res..

[104] Mousavi S.M., Milajerdi A., Sheikhi A., Kord-Varkaneh H., Feinle-Bisset C., Larijani B., Esmaillzadeh A. (2019). Resveratrol supplementation significantly influences obesity measures: A systematic review and dose-response meta-analysis of randomized controlled trials. Obes. Rev..

[105] Truong V.L., Jun M., Jeong W.S. (2018). Role of resveratrol in regulation of cellular defense systems against oxidative stress. Biofactors.

[106] Cao D., Wang M., Qiu X., Liu D., Jiang H., Yang N., Xu R.M. (2015). Structural basis for allosteric, substrate-dependent stimulation of SIRT1 activity by resveratrol. Genes. Dev..

[107] Palacios J.A., Herranz D., De Bonis M.L., Velasco S., Serrano M., Blasco M.A. (2010). SIRT1 contributes to telomere maintenance and augments global homologous recombination. J. Cell Biol..

[108] Xia L., Wang X.X., Hu X.S., Guo X.G., Shang Y.P., Chen H.J., Zeng C.L., Zhang F.R., Chen J.Z. (2008). Resveratrol reduces endothelial progenitor cells senescence through augmentation of telomerase activity by Akt-dependent mechanisms. Br. J. Pharmacol..

[109] del Bas J.M., Crescenti A., Arola-Arnal A., Oms-Oliu G., Arola L., Caimari A. (2015). Grape seed procyanidin supplementation to rats fed a high-fat diet during pregnancy and lactation increases the body fat content and modulates the inflammatory response and the adipose tissue metabolism of the male offspring in youth. Int. J. Obes..

[110] Zou T., Chen D., Yang Q., Wang B., Zhu M.J., Nathanielsz P.W., Du M. (2017). Resveratrol supplementation of high-fat diet-fed pregnant mice promotes brown and beige adipocyte development and prevents obesity in male offspring. J. Physiol..

[111] Liu T.Y., Yu H.R., Tsai C.C., Huang L.T., Chen C.C., Sheen J.M., Tiao M.M., Tain Y.L., Lin I.C., Lai Y.J. (2020). Resveratrol intake during pregnancy and lactation re-programs adiposity and ameliorates leptin resistance in male progeny induced by maternal high-fat/high sucrose plus postnatal high-fat/high sucrose diets via fat metabolism regulation. Lipids Health Dis..

[112] Tanaka M., Kita T., Yamasaki S., Kawahara T., Ueno Y., Yamada M., Mukai Y., Sato S., Kurasaki M., Saito T. (2017). Maternal resveratrol intake during lactation attenuates hepatic triglyceride and fatty acid synthesis in adult male rat offspring. Biochem. Biophys. Rep..

[113] Caimari A., Marine-Casado R., Boque N., Crescenti A., Arola L., Del Bas J.M. (2017). Maternal intake of grape seed procyanidins during lactation induces insulin resistance and an adiponectin resistance-like phenotype in rat offspring. Sci. Rep..

[114] Bourque S.L., Dolinsky V.W., Dyck J.R., Davidge S.T. (2012). Maternal resveratrol treatment during pregnancy improves adverse fetal outcomes in a rat model of severe hypoxia. Placenta.

[115] Arola-Arnal A., Oms-Oliu G., Crescenti A., del Bas J.M., Ras M.R., Arola L., Caimari A. (2013). Distribution of grape seed flavanols and their metabolites in pregnant rats and their fetuses. Mol. Nutr. Food Res..

[116] Serrano A., Ribot J., Palou A., Bonet M.L. (2021). Long-term programming of skeletal muscle and liver lipid and energy metabolism by resveratrol supplementation to suckling mice. J. Nutr. Biochem..

[117] Kataoka S., Norikura T., Sato S. (2018). Maternal green tea polyphenol intake during lactation attenuates kidney injury in high-fat-diet-fed male offspring programmed by maternal protein restriction in rats. J. Nutr. Biochem..

[118] Price N.L., Gomes A.P., Ling A.J., Duarte F.V., Martin-Montalvo A., North B.J., Agarwal B., Ye L., Ramadori G., Teodoro J.S. (2012). SIRT1 is required for AMPK activation and the beneficial effects of resveratrol on mitochondrial function. Cell Metab..

[119] Lu Z., Chan Y.T., Lo K.K., Wong V.W., Ng Y.F., Li S.Y., Ho W.W., Wong M.S., Zhao D. (2021). Levels of polyphenols and phenolic metabolites in breast milk and their association with plant-based food intake in Hong Kong lactating women. Food Funct..

[120] Lopez-Yerena A., Grases-Pinto B., Zhan-Dai S., Perez-Cano F.J., Lamuela-Raventos R.M., Rodriguez-Lagunas M.J., Vallverdu-Queralt A. (2022). Nutrition during pregnancy and lactation: New evidence for the vertical transmission of extra virgin olive oil phenolic compounds in rats. Food Chem..

[121] Henning S.M., Yang J., Lee R.P., Huang J., Thames G., Korn M., Ben-Nissan D., Heber D., Li Z. (2022). Pomegranate juice alters the microbiota in breast milk and infant stool: A pilot study. Food Funct..

[122] Reynes B., Palou M., Rodriguez A.M., Palou A. (2018). Regulation of Adaptive Thermogenesis and Browning by Prebiotics and Postbiotics. Front. Physiol..

[123] Du S., Jin J., Fang W., Su Q. (2015). Does Fish Oil Have an Anti-Obesity Effect in Overweight/Obese Adults? A Meta-Analysis of Randomized Controlled Trials. PLoS ONE.

[124] Okla M., Kim J., Koehler K., Chung S. (2017). Dietary Factors Promoting Brown and Beige Fat Development and Thermogenesis. Adv. Nutr..

[125] Kim J., Okla M., Erickson A., Carr T., Natarajan S.K., Chung S. (2016). Eicosapentaenoic Acid Potentiates Brown Thermogenesis through FFAR4-dependent Up-regulation of miR-30b and miR-378. J. Biol. Chem..

[126] Quesada-Lopez T., Cereijo R., Turatsinze J.V., Planavila A., Cairo M., Gavalda-Navarro A., Peyrou M., Moure R., Iglesias R., Giralt M. (2016). The lipid sensor GPR120 promotes brown fat activation and FGF21 release from adipocytes. Nat. Commun..

[127] Owen B.M., Ding X., Morgan D.A., Coate K.C., Bookout A.L., Rahmouni K., Kliewer S.A., Mangelsdorf D.J. (2014). FGF21 acts centrally to induce sympathetic nerve activity, energy expenditure, and weight loss. Cell Metab..

[128] Yang F., Zhou N., Zhu X., Min C., Zhou W., Li X. (2021). n-3 PUFAs protect against adiposity and fatty liver by promoting browning in postnatally overfed male rats: A role for NRG4. J. Nutr. Biochem..

[129] Liu Y., Chen M. (2022). Neuregulin 4 as a novel adipokine in energy metabolism. Front. Physiol..

[130] Comas F., Martinez C., Sabater M., Ortega F., Latorre J., Diaz-Saez F., Aragones J., Camps M., Guma A., Ricart W. (2019). Neuregulin 4 Is a Novel Marker of Beige Adipocyte Precursor Cells in Human Adipose Tissue. Front. Physiol..

[131] Kim M., Goto T., Yu R., Uchida K., Tominaga M., Kano Y., Takahashi N., Kawada T. (2015). Fish oil intake induces UCP1 upregulation in brown and white adipose tissue via the sympathetic nervous system. Sci. Rep..

[132] Ailhaud G., Massiera F., Weill P., Legrand P., Alessandri J.M., Guesnet P. (2006). Temporal changes in dietary fats: Role of n-6 polyunsaturated fatty acids in excessive adipose tissue development and relationship to obesity. Prog. Lipid Res..

[133] Massiera F., Saint-Marc P., Seydoux J., Murata T., Kobayashi T., Narumiya S., Guesnet P., Amri E.Z., Negrel R., Ailhaud G. (2003). Arachidonic acid and prostacyclin signaling promote adipose tissue development: A human health concern?. J. Lipid Res..

[134] Kim H.K., Della-Fera M., Lin J., Baile C.A. (2006). Docosahexaenoic acid inhibits adipocyte differentiation and induces apoptosis in 3T3-L1 preadipocytes. J. Nutr..

[135] Ailhaud G., Guesnet P. (2004). Fatty acid composition of fats is an early determinant of childhood obesity: A short review and an opinion. Obes. Rev..

[136] Muhlhausler B.S., Ailhaud G.P. (2013). Omega-6 polyunsaturated fatty acids and the early origins of obesity. Curr. Opin. Endocrinol. Diabetes Obes..

[137] Massiera F., Barbry P., Guesnet P., Joly A., Luquet S., Moreilhon-Brest C., Mohsen-Kanson T., Amri E.Z., Ailhaud G. (2010). A Western-like fat diet is sufficient to induce a gradual enhancement in fat mass over generations. J. Lipid Res..

[138] Miliku K., Duan Q.L., Moraes T.J., Becker A.B., Mandhane P.J., Turvey S.E., Lefebvre D.L., Sears M.R., Subbarao P., Field C.J. (2019). Human milk fatty acid composition is associated with dietary, genetic, sociodemographic, and environmental factors in the CHILD Cohort Study. Am. J. Clin. Nutr..

[139] Donahue S.M., Rifas-Shiman S.L., Gold D.R., Jouni Z.E., Gillman M.W., Oken E. (2011). Prenatal fatty acid status and child adiposity at age 3 y: Results from a US pregnancy cohort. Am. J. Clin. Nutr..

[140] Rudolph M.C., Young B.E., Lemas D.J., Palmer C.E., Hernandez T.L., Barbour L.A., Friedman J.E., Krebs N.F., MacLean P.S. (2017). Early infant adipose deposition is positively associated with the n-6 to n-3 fatty acid ratio in human milk independent of maternal BMI. Int. J. Obes..

[141] Chilton F.H., Murphy R.C., Wilson B.A., Sergeant S., Ainsworth H., Seeds M.C., Mathias R.A. (2014). Diet-gene interactions and PUFA metabolism: A potential contributor to health disparities and human diseases. Nutrients.

[142] Rudolph M.C., Jackman M.R., Presby D.M., Houck J.A., Webb P.G., Johnson G.C., Soderborg T.K., de la Houssaye B.A., Yang I.V., Friedman J.E. (2018). Low Neonatal Plasma n-6/n-3 PUFA Ratios Regulate Offspring Adipogenic Potential and Condition Adult Obesity Resistance. Diabetes.

[143] Heerwagen M.J., Stewart M.S., de la Houssaye B.A., Janssen R.C., Friedman J.E. (2013). Transgenic increase in N-3/n-6 Fatty Acid ratio reduces maternal obesity-associated inflammation and limits adverse developmental programming in mice. PLoS ONE.

[144] Fan R., Toney A.M., Jang Y., Ro S.H., Chung S. (2018). Maternal n-3 PUFA supplementation promotes fetal brown adipose tissue development through epigenetic modifications in C57BL/6 mice. Biochim. Biophys. Acta. Mol. Cell Biol. Lipids.

[145] Segovia S.A., Vickers M.H., Zhang X.D., Gray C., Reynolds C.M. (2015). Maternal supplementation with conjugated linoleic acid in the setting of diet-induced obesity normalises the inflammatory phenotype in mothers and reverses metabolic dysfunction and impaired insulin sensitivity in offspring. J. Nutr. Biochem..

[146] Reynolds C.M., Segovia S.A., Zhang X.D., Gray C., Vickers M.H. (2015). Conjugated linoleic Acid supplementation during pregnancy and lactation reduces maternal high-fat-diet-induced programming of early-onset puberty and hyperlipidemia in female rat offspring. Biol. Reprod..

[147] Segovia S.A., Vickers M.H., Gray C., Zhang X.D., Reynolds C.M. (2017). Conjugated Linoleic Acid Supplementation Improves Maternal High Fat Diet-Induced Programming of Metabolic Dysfunction in Adult Male Rat Offspring. Sci. Rep..

[148] Weber J.A., Baxter D.H., Zhang S., Huang D.Y., Huang K.H., Lee M.J., Galas D.J., Wang K. (2010). The microRNA spectrum in 12 body fluids. Clin. Chem..

[149] Izumi H., Kosaka N., Shimizu T., Sekine K., Ochiya T., Takase M. (2012). Bovine milk contains microRNA and messenger RNA that are stable under degradative conditions. J. Dairy. Sci..

[150] Baier S.R., Nguyen C., Xie F., Wood J.R., Zempleni J. (2014). MicroRNAs are absorbed in biologically meaningful amounts from nutritionally relevant doses of cow milk and affect gene expression in peripheral blood mononuclear cells, HEK-293 kidney cell cultures, and mouse livers. J. Nutr..

[151] Floris I., Kraft J.D., Altosaar I. (2016). Roles of MicroRNA across Prenatal and Postnatal Periods. Int. J. Mol. Sci..

[152] Leroux C., Chervet M.L., German J.B. (2021). Perspective: Milk microRNAs as Important Players in Infant Physiology and Development. Adv. Nutr..

[153] Carrillo-Lozano E., Sebastian-Valles F., Knott-Torcal C. (2020). Circulating microRNAs in Breast Milk and Their Potential Impact on the Infant. Nutrients.

[154] Hatmal M.M., Al-Hatamleh M.A.I., Olaimat A.N., Alshaer W., Hasan H., Albakri K.A., Alkhafaji E., Issa N.N., Al-Holy M.A., Abderrahman S.M. (2022). Immunomodulatory Properties of Human Breast Milk: MicroRNA Contents and Potential Epigenetic Effects. Biomedicines.

[155] Gialeli G., Panagopoulou O., Liosis G., Siahanidou T. (2023). Potential Epigenetic Effects of Human Milk on Infants’ Neurodevelopment. Nutrients.

[156] Melnik B.C., Schmitz G. (2017). MicroRNAs: Milk’s epigenetic regulators. Best. Pract. Res. Clin. Endocrinol. Metab..

[157] Melnik B.C., Stremmel W., Weiskirchen R., John S.M., Schmitz G. (2021). Exosome-Derived MicroRNAs of Human Milk and Their Effects on Infant Health and Development. Biomolecules.

[158] Abbas M.A., Al-Saigh N.N., Saqallah F.G. (2023). Regulation of adipogenesis by exosomal milk miRNA. Rev. Endocr. Metab. Disord..

[159] Kurylowicz A. (2021). microRNAs in Human Adipose Tissue Physiology and Dysfunction. Cells.

[160] Gharanei S., Shabir K., Brown J.E., Weickert M.O., Barber T.M., Kyrou I., Randeva H.S. (2020). Regulatory microRNAs in Brown, Brite and White Adipose Tissue. Cells.

[161] Pomar C.A., Castro H., Pico C., Serra F., Palou A., Sanchez J. (2019). Cafeteria Diet Consumption during Lactation in Rats, Rather than Obesity Per Se, alters miR-222, miR-200a, and miR-26a Levels in Milk. Mol. Nutr. Food Res..

[162] Zamanillo R., Sanchez J., Serra F., Palou A. (2019). Breast Milk Supply of MicroRNA Associated with Leptin and Adiponectin Is Affected by Maternal Overweight/Obesity and Influences Infancy BMI. Nutrients.

[163] Alonso-Bernaldez M., Asensio A., Palou-March A., Sanchez J., Palou A., Serra F., Palou M. (2022). Breast Milk MicroRNAs Related to Leptin and Adiponectin Function Can Be Modulated by Maternal Diet and Influence Offspring Phenotype in Rats. Int. J. Mol. Sci..

[164] Bibiloni P., Pomar C.A., Palou A., Sanchez J., Serra F. (2023). miR-222 exerts negative regulation on insulin signaling pathway in 3T3-L1 adipocytes. Biofactors.

[165] Pomar C.A., Serra F., Palou A., Sanchez J. (2021). Lower miR-26a levels in breastmilk affect gene expression in adipose tissue of offspring. FASEB J..

[166] Lowry D.E., Paul H.A., Reimer R.A. (2022). Impact of maternal obesity and prebiotic supplementation on select maternal milk microRNA levels and correlation with offspring outcomes. Br. J. Nutr..

[167] Xi Y., Jiang X., Li R., Chen M., Song W., Li X. (2016). The levels of human milk microRNAs and their association with maternal weight characteristics. Eur. J. Clin. Nutr..

[168] Shah K.B., Chernausek S.D., Garman L.D., Pezant N.P., Plows J.F., Kharoud H.K., Demerath E.W., Fields D.A. (2021). Human Milk Exosomal MicroRNA: Associations with Maternal Overweight/Obesity and Infant Body Composition at 1 Month of Life. Nutrients.

[169] Shah K.B., Fields D.A., Pezant N.P., Kharoud H.K., Gulati S., Jacobs K., Gale C.A., Kharbanda E.O., Nagel E.M., Demerath E.W. (2022). Gestational Diabetes Mellitus Is Associated with Altered Abundance of Exosomal MicroRNAs in Human Milk. Clin. Ther..

[170] Zhou Q., Li M., Wang X., Li Q., Wang T., Zhu Q., Zhou X., Wang X., Gao X., Li X. (2012). Immune-related microRNAs are abundant in breast milk exosomes. Int. J. Biol. Sci..

[171] Shi C., Zhang M., Tong M., Yang L., Pang L., Chen L., Xu G., Chi X., Hong Q., Ni Y. (2015). miR-148a is Associated with Obesity and Modulates Adipocyte Differentiation of Mesenchymal Stem Cells through Wnt Signaling. Sci. Rep..

[172] He H., Cai M., Zhu J., Xiao W., Liu B., Shi Y., Yang X., Liang X., Zheng T., Hu S. (2018). miR-148a-3p promotes rabbit preadipocyte differentiation by targeting PTEN. In Vitro Cell Dev. Biol. Anim..

[173] Jin X., Hao Z., Zhao M., Shen J., Ke N., Song Y., Qiao L., Lu Y., Hu L., Wu X. (2021). MicroRNA-148a Regulates the Proliferation and Differentiation of Ovine Preadipocytes by Targeting PTEN. Animals.

[174] Duan D.Y., Tang J., Tian H.T., Shi Y.Y., Jia J. (2021). Adipocyte-secreted microvesicle-derived miR-148a regulates adipogenic and osteogenic differentiation by targeting Wnt5a/Ror2 pathway. Life Sci..

[175] Cho Y.M., Kim T.M., Hun Kim D., Hee Kim D., Jeong S.W., Kwon O.J. (2016). miR-148a is a downstream effector of X-box-binding protein 1 that silences Wnt10b during adipogenesis of 3T3-L1 cells. Exp. Mol. Med..

[176] Shore A., Karamitri A., Kemp P., Speakman J.R., Lomax M.A. (2010). Role of Ucp1 enhancer methylation and chromatin remodelling in the control of Ucp1 expression in murine adipose tissue. Diabetologia.

[177] Hu F., Wang M., Xiao T., Yin B., He L., Meng W., Dong M., Liu F. (2015). miR-30 promotes thermogenesis and the development of beige fat by targeting RIP140. Diabetes.

[178] Li G., Wu Z., Li X., Ning X., Li Y., Yang G. (2011). Biological role of microRNA-103 based on expression profile and target genes analysis in pigs. Mol. Biol. Rep..

[179] Li M., Liu Z., Zhang Z., Liu G., Sun S., Sun C. (2015). miR-103 promotes 3T3-L1 cell adipogenesis through AKT/mTOR signal pathway with its target being MEF2D. Biol. Chem..

[180] Zhang Z., Wu S., Muhammad S., Ren Q., Sun C. (2018). miR-103/107 promote ER stress-mediated apoptosis via targeting the Wnt3a/beta-catenin/ATF6 pathway in preadipocytes. J. Lipid Res..

[181] Alkhouri N., Gornicka A., Berk M.P., Thapaliya S., Dixon L.J., Kashyap S., Schauer P.R., Feldstein A.E. (2010). Adipocyte apoptosis, a link between obesity, insulin resistance, and hepatic steatosis. J. Biol. Chem..

[182] Trajkovski M., Hausser J., Soutschek J., Bhat B., Akin A., Zavolan M., Heim M.H., Stoffel M. (2011). MicroRNAs 103 and 107 regulate insulin sensitivity. Nature.

[183] McGregor R.A., Choi M.S. (2011). microRNAs in the regulation of adipogenesis and obesity. Curr. Mol. Med..

[184] Zhang C., Qian D., Zhao H., Lv N., Yu P., Sun Z. (2018). MiR17 improves insulin sensitivity through inhibiting expression of ASK1 and anti-inflammation of macrophages. Biomed. Pharmacother..

[185] Li H., Chen X., Guan L., Qi Q., Shu G., Jiang Q., Yuan L., Xi Q., Zhang Y. (2013). MiRNA-181a regulates adipogenesis by targeting tumor necrosis factor-alpha (TNF-alpha) in the porcine model. PLoS ONE.

[186] Chartoumpekis D.V., Zaravinos A., Ziros P.G., Iskrenova R.P., Psyrogiannis A.I., Kyriazopoulou V.E., Habeos I.G. (2012). Differential expression of microRNAs in adipose tissue after long-term high-fat diet-induced obesity in mice. PLoS ONE.

[187] Herrera B.M., Lockstone H.E., Taylor J.M., Ria M., Barrett A., Collins S., Kaisaki P., Argoud K., Fernandez C., Travers M.E. (2010). Global microRNA expression profiles in insulin target tissues in a spontaneous rat model of type 2 diabetes. Diabetologia.

[188] Ortega F.J., Mercader J.M., Catalan V., Moreno-Navarrete J.M., Pueyo N., Sabater M., Gomez-Ambrosi J., Anglada R., Fernandez-Formoso J.A., Ricart W. (2013). Targeting the circulating microRNA signature of obesity. Clin. Chem..

[189] Cui X., You L., Zhu L., Wang X., Zhou Y., Li Y., Wen J., Xia Y., Wang X., Ji C. (2018). Change in circulating microRNA profile of obese children indicates future risk of adult diabetes. Metabolism.

[190] Ortega F.J., Mercader J.M., Moreno-Navarrete J.M., Rovira O., Guerra E., Esteve E., Xifra G., Martinez C., Ricart W., Rieusset J. (2014). Profiling of circulating microRNAs reveals common microRNAs linked to type 2 diabetes that change with insulin sensitization. Diabetes Care.

[191] Giardina S., Hernandez-Alonso P., Diaz-Lopez A., Salas-Huetos A., Salas-Salvado J., Bullo M. (2019). Changes in circulating miRNAs in healthy overweight and obese subjects: Effect of diet composition and weight loss. Clin. Nutr..

[192] Ahn J., Lee H., Jung C.H., Jeon T.I., Ha T.Y. (2013). MicroRNA-146b promotes adipogenesis by suppressing the SIRT1-FOXO1 cascade. EMBO Mol. Med..

[193] Pan X.X., Cao J.M., Cai F., Ruan C.C., Wu F., Gao P.J. (2018). Loss of miR-146b-3p Inhibits Perivascular Adipocyte Browning with Cold Exposure During Aging. Cardiovasc. Drugs Ther..

[194] Pomar C.A., van Nes R., Sanchez J., Pico C., Keijer J., Palou A. (2017). Maternal consumption of a cafeteria diet during lactation in rats leads the offspring to a thin-outside-fat-inside phenotype. Int. J. Obes..

[195] Pomar C.A., Kuda O., Kopecky J., Rombaldova M., Castro H., Pico C., Sanchez J., Palou A. (2019). Alterations in plasma acylcarnitine and amino acid profiles may indicate poor nutrition during the suckling period due to maternal intake of an unbalanced diet and may predict later metabolic dysfunction. FASEB J..

[196] Pomar C.A., Castillo P., Palou A., Palou M., Pico C. (2022). Dietary Improvement during Lactation Normalizes miR-26a, miR-222 and miR-484 Levels in the Mammary Gland, but Not in Milk, of Diet-Induced Obese Rats. Biomedicines.

[197] Castillo P., Pomar C.A., Palou A., Palou M., Pico C. (2023). Influence of Maternal Metabolic Status and Diet during the Perinatal Period on the Metabolic Programming by Leptin Ingested during the Suckling Period in Rats. Nutrients.

[198] Castillo P., Palou M., Otero D., Nunez P., Palou A., Pico C. (2021). Sex-Specific Effects of Myo-Inositol Ingested During Lactation in the Improvement of Metabolic Health in Adult Rats. Mol. Nutr. Food Res..

[199] Badillo-Suarez P.A., Rodriguez-Cruz M., Nieves-Morales X. (2017). Impact of Metabolic Hormones Secreted in Human Breast Milk on Nutritional Programming in Childhood Obesity. J. Mammary Gland. Biol. Neoplasia.

[200] Kratzsch J., Bae Y.J., Kiess W. (2018). Adipokines in human breast milk. Best. Pract. Res. Clin. Endocrinol. Metab..

[201] Pico C., Oliver P., Sanchez J., Miralles O., Caimari A., Priego T., Palou A. (2007). The intake of physiological doses of leptin during lactation in rats prevents obesity in later life. Int. J. Obes..

[202] Palou M., Pico C., Palou A. (2018). Leptin as a breast milk component for the prevention of obesity. Nutr. Rev..

[203] Zhang Y., Proenca R., Maffei M., Barone M., Leopold L., Friedman J.M. (1994). Positional cloning of the mouse obese gene and its human homologue. Nature.

[204] Ahima R.S., Flier J.S. (2000). Leptin. Annu. Rev. Physiol..

[205] Pico C., Palou M., Pomar C.A., Rodriguez A.M., Palou A. (2022). Leptin as a key regulator of the adipose organ. Rev. Endocr. Metab. Disord..

[206] Smith-Kirwin S.M., O’Connor D.M., De Johnston J., Lancey E.D., Hassink S.G., Funanage V.L. (1998). Leptin expression in human mammary epithelial cells and breast milk. J. Clin. Endocrinol. Metab..

[207] Casabiell X., Pineiro V., Tome M.A., Peino R., Dieguez C., Casanueva F.F. (1997). Presence of leptin in colostrum and/or breast milk from lactating mothers: A potential role in the regulation of neonatal food intake. J. Clin. Endocrinol. Metab..

[208] Houseknecht K.L., McGuire M.K., Portocarrero C.P., McGuire M.A., Beerman K. (1997). Leptin is present in human milk and is related to maternal plasma leptin concentration and adiposity. Biochem. Biophys. Res. Commun..

[209] O’Connor D., Funanage V., Locke R., Spear M., Leef K. (2003). Leptin is not present in infant formulas. J. Endocrinol. Investig..

[210] Palou A., Picó C., Oliver P., Sánchez J., Miralles O. (2005). Use of Leptin for the Prevention of Excess Body Weight and Composition Containing Leptin. U.S. Patent.

[211] Miralles O., Sanchez J., Palou A., Pico C. (2006). A physiological role of breast milk leptin in body weight control in developing infants. Obesity.

[212] Doneray H., Orbak Z., Yildiz L. (2009). The relationship between breast milk leptin and neonatal weight gain. Acta Paediatr..

[213] Schuster S., Hechler C., Gebauer C., Kiess W., Kratzsch J. (2011). Leptin in maternal serum and breast milk: Association with infants’ body weight gain in a longitudinal study over 6 months of lactation. Pediatr. Res..

[214] Fields D.A., Demerath E.W. (2012). Relationship of insulin, glucose, leptin, IL-6 and TNF-alpha in human breast milk with infant growth and body composition. Pediatr. Obes..

[215] Chan D., Goruk S., Becker A.B., Subbarao P., Mandhane P.J., Turvey S.E., Lefebvre D., Sears M.R., Field C.J., Azad M.B. (2018). Adiponectin, leptin and insulin in breast milk: Associations with maternal characteristics and infant body composition in the first year of life. Int. J. Obes..

[216] Weyermann M., Brenner H., Rothenbacher D. (2007). Adipokines in human milk and risk of overweight in early childhood: A prospective cohort study. Epidemiology.

[217] Kon I.Y., Shilina N.M., Gmoshinskaya M.V., Ivanushkina T.A. (2014). The study of breast milk IGF-1, leptin, ghrelin and adiponectin levels as possible reasons of high weight gain in breast-fed infants. Ann. Nutr. Metab..

[218] Brunner S., Schmid D., Zang K., Much D., Knoeferl B., Kratzsch J., Amann-Gassner U., Bader B.L., Hauner H. (2015). Breast milk leptin and adiponectin in relation to infant body composition up to 2 years. Pediatr. Obes..

[219] Meyer D.M., Brei C., Stecher L., Much D., Brunner S., Hauner H. (2017). The relationship between breast milk leptin and adiponectin with child body composition from 3 to 5 years: A follow-up study. Pediatr. Obes..

[220] Sanchez J., Priego T., Palou M., Tobaruela A., Palou A., Pico C. (2008). Oral supplementation with physiological doses of leptin during lactation in rats improves insulin sensitivity and affects food preferences later in life. Endocrinology.

[221] Priego T., Sanchez J., Palou A., Pico C. (2010). Leptin intake during the suckling period improves the metabolic response of adipose tissue to a high-fat diet. Int. J. Obes..

[222] Bouret S.G., Draper S.J., Simerly R.B. (2004). Trophic action of leptin on hypothalamic neurons that regulate feeding. Science.

[223] Pico C., Palou M., Priego T., Sanchez J., Palou A. (2012). Metabolic programming of obesity by energy restriction during the perinatal period: Different outcomes depending on gender and period, type and severity of restriction. Front. Physiol..

[224] Palou M., Konieczna J., Torrens J.M., Sanchez J., Priego T., Fernandes M.L., Palou A., Pico C. (2012). Impaired insulin and leptin sensitivity in the offspring of moderate caloric-restricted dams during gestation is early programmed. J. Nutr. Biochem..

[225] Konieczna J., Garcia A.P., Sanchez J., Palou M., Palou A., Pico C. (2013). Oral leptin treatment in suckling rats ameliorates detrimental effects in hypothalamic structure and function caused by maternal caloric restriction during gestation. PLoS ONE.

[226] Castillo P., Palou M., Yau-Qiu Z.X., Rodriguez A.M., Palou A., Pico C. (2021). Myo-Inositol Supplementation in Suckling Rats Protects against Adverse Programming Outcomes on Hypothalamic Structure Caused by Mild Gestational Calorie Restriction, Partially Comparable to Leptin Effects. Nutrients.

[227] Szostaczuk N., Priego T., Palou M., Palou A., Pico C. (2017). Oral leptin supplementation throughout lactation in rats prevents later metabolic alterations caused by gestational calorie restriction. Int. J. Obes..

[228] Candler T., Kuhnen P., Prentice A.M., Silver M. (2019). Epigenetic regulation of POMC; implications for nutritional programming, obesity and metabolic disease. Front. Neuroendocrinol..

[229] Palou M., Pico C., McKay J.A., Sanchez J., Priego T., Mathers J.C., Palou A. (2011). Protective effects of leptin during the suckling period against later obesity may be associated with changes in promoter methylation of the hypothalamic pro-opiomelanocortin gene. Br. J. Nutr..

[230] Palou A., Pico C., Bonet M.L. (2013). Nutritional potential of metabolic remodelling of white adipose tissue. Curr. Opin. Clin. Nutr. Metab. Care.

[231] Garcia A.P., Palou M., Sanchez J., Priego T., Palou A., Pico C. (2011). Moderate caloric restriction during gestation in rats alters adipose tissue sympathetic innervation and later adiposity in offspring. PLoS ONE.

[232] Palou M., Priego T., Romero M., Szostaczuk N., Konieczna J., Cabrer C., Remesar X., Palou A., Pico C. (2015). Moderate calorie restriction during gestation programs offspring for lower BAT thermogenic capacity driven by thyroid and sympathetic signaling. Int. J. Obes..

[233] Youngstrom T.G., Bartness T.J. (1998). White adipose tissue sympathetic nervous system denervation increases fat pad mass and fat cell number. Am. J. Physiol..

[234] Konieczna J., Palou M., Sanchez J., Pico C., Palou A. (2015). Leptin intake in suckling rats restores altered T3 levels and markers of adipose tissue sympathetic drive and function caused by gestational calorie restriction. Int. J. Obes..

[235] Commins S.P., Watson P.M., Padgett M.A., Dudley A., Argyropoulos G., Gettys T.W. (1999). Induction of uncoupling protein expression in brown and white adipose tissue by leptin. Endocrinology.

[236] Scarpace P.J., Matheny M. (1998). Leptin induction of UCP1 gene expression is dependent on sympathetic innervation. Am. J. Physiol..

[237] Plum L., Rother E., Munzberg H., Wunderlich F.T., Morgan D.A., Hampel B., Shanabrough M., Janoschek R., Konner A.C., Alber J. (2007). Enhanced leptin-stimulated Pi3k activation in the CNS promotes white adipose tissue transdifferentiation. Cell Metab..

[238] Siegrist-Kaiser C.A., Pauli V., Juge-Aubry C.E., Boss O., Pernin A., Chin W.W., Cusin I., Rohner-Jeanrenaud F., Burger A.G., Zapf J. (1997). Direct effects of leptin on brown and white adipose tissue. J. Clin. Investig..

[239] Pucci E., Chiovato L., Pinchera A. (2000). Thyroid and lipid metabolism. Int. J. Obes. Relat. Metab. Disord..

[240] Sentis S.C., Oelkrug R., Mittag J. (2021). Thyroid hormones in the regulation of brown adipose tissue thermogenesis. Endocr. Connect..

[241] Vickers M.H., Gluckman P.D., Coveny A.H., Hofman P.L., Cutfield W.S., Gertler A., Breier B.H., Harris M. (2008). The effect of neonatal leptin treatment on postnatal weight gain in male rats is dependent on maternal nutritional status during pregnancy. Endocrinology.

[242] Vickers M.H., Gluckman P.D., Coveny A.H., Hofman P.L., Cutfield W.S., Gertler A., Breier B.H., Harris M. (2005). Neonatal leptin treatment reverses developmental programming. Endocrinology.

[243] Fruhbeck G., Catalan V., Rodriguez A., Gomez-Ambrosi J. (2018). Adiponectin-leptin ratio: A promising index to estimate adipose tissue dysfunction. Relation with obesity-associated cardiometabolic risk. Adipocyte.

[244] Fruhbeck G., Catalan V., Rodriguez A., Ramirez B., Becerril S., Salvador J., Colina I., Gomez-Ambrosi J. (2019). Adiponectin-leptin Ratio is a Functional Biomarker of Adipose Tissue Inflammation. Nutrients.

[245] Tessier D.R., Ferraro Z.M., Gruslin A. (2013). Role of leptin in pregnancy: Consequences of maternal obesity. Placenta.

[246] Yau-Qiu Z.X., Pico C., Rodriguez A.M., Palou A. (2020). Leptin Distribution in Rat Foetal and Extraembryonic Tissues in Late Gestation: A Physiological View of Amniotic Fluid Leptin. Nutrients.

[247] Katan M., Cockcroft S. (2020). Phosphatidylinositol(4,5)bisphosphate: Diverse functions at the plasma membrane. Essays Biochem..

[248] Zhao H., Xing C., Zhang J., He B. (2021). Comparative efficacy of oral insulin sensitizers metformin, thiazolidinediones, inositol, and berberine in improving endocrine and metabolic profiles in women with PCOS: A network meta-analysis. Reprod. Health.

[249] DiNicolantonio J.J., O’Keefe J.H. (2022). Myo-inositol for insulin resistance, metabolic syndrome, polycystic ovary syndrome and gestational diabetes. Open Heart.

[250] Ortmeyer H.K. (1996). Dietary myoinositol results in lower urine glucose and in lower postprandial plasma glucose in obese insulin resistant rhesus monkeys. Obes. Res..

[251] Dang N.T., Mukai R., Yoshida K., Ashida H. (2010). D-pinitol and myo-inositol stimulate translocation of glucose transporter 4 in skeletal muscle of C57BL/6 mice. Biosci. Biotechnol. Biochem..

[252] Croze M.L., Vella R.E., Pillon N.J., Soula H.A., Hadji L., Guichardant M., Soulage C.O. (2013). Chronic treatment with myo-inositol reduces white adipose tissue accretion and improves insulin sensitivity in female mice. J. Nutr. Biochem..

[253] Santamaria A., Giordano D., Corrado F., Pintaudi B., Interdonato M.L., Vieste G.D., Benedetto A.D., D’Anna R. (2012). One-year effects of myo-inositol supplementation in postmenopausal women with metabolic syndrome. Climacteric.

[254] Mashayekh-Amiri S., Mohammad-Alizadeh-Charandabi S., Abdolalipour S., Mirghafourvand M. (2022). Myo-inositol supplementation for prevention of gestational diabetes mellitus in overweight and obese pregnant women: A systematic review and meta-analysis. Diabetol. Metab. Syndr..

[255] Factor P.A., Corpuz H. (2023). The Efficacy and Safety of Myo-inositol Supplementation for the Prevention of Gestational Diabetes Mellitus in Overweight and Obese Pregnant Women: A Systematic Review and Meta-Analysis. J. ASEAN Fed. Endocr. Soc..

[256] Palou M., Torrens J.M., Castillo P., Sanchez J., Palou A., Pico C. (2020). Metabolomic approach in milk from calorie-restricted rats during lactation: A potential link to the programming of a healthy phenotype in offspring. Eur. J. Nutr..

[257] Bromberger P., Hallman M. (1986). Myoinositol in small preterm infants: Relationship between intake and serum concentration. J. Pediatr. Gastroenterol. Nutr..

[258] Pereira G.R., Baker L., Egler J., Corcoran L., Chiavacci R. (1990). Serum myoinositol concentrations in premature infants fed human milk, formula for infants, and parenteral nutrition. Am. J. Clin. Nutr..

[259] He X., Parenti M., Grip T., Domellof M., Lonnerdal B., Hernell O., Timby N., Slupsky C.M. (2019). Metabolic phenotype of breast-fed infants, and infants fed standard formula or bovine MFGM supplemented formula: A randomized controlled trial. Sci. Rep..

[260] Palou M., Priego T., Sanchez J., Torrens J.M., Palou A., Pico C. (2010). Moderate caloric restriction in lactating rats protects offspring against obesity and insulin resistance in later life. Endocrinology.

[261] Palou M., Torrens J.M., Priego T., Sanchez J., Palou A., Pico C. (2011). Moderate caloric restriction in lactating rats programs their offspring for a better response to HF diet feeding in a sex-dependent manner. J. Nutr. Biochem..

[262] Valle A., Castillo P., García-Rodríguez A., Palou M., Palou A., Pico C. (2024). BDNF as a potential mediator of the beneficial effects of myo-inositol supplementation during suckling in the offspring of gestational calorie-restricted rats. Nutrients.

[263] Di Rosa M.C., Zimbone S., Saab M.W., Tomasello M.F. (2021). The Pleiotropic Potential of BDNF beyond Neurons: Implication for a Healthy Mind in a Healthy Body. Life.

[264] Larsen L.H., Sando-Pedersen S., Orstrup L.K.H., Grunnet N., Quistorff B., Mortensen O.H. (2017). Gestational Protein Restriction in Wistar Rats; Effect of Taurine Supplementation on Properties of Newborn Skeletal Muscle. Adv. Exp. Med. Biol..

[265] Li M., Reynolds C.M., Sloboda D.M., Gray C., Vickers M.H. (2015). Maternal taurine supplementation attenuates maternal fructose-induced metabolic and inflammatory dysregulation and partially reverses adverse metabolic programming in offspring. J. Nutr. Biochem..

[266] Cetin A.K., Buyukdere Y., Gulec A., Akyol A. (2023). Taurine supplementation reduces adiposity and hepatic lipid metabolic activity in adult offspring following maternal cafeteria diet. Nutr. Res..

[267] Bay J.L., Morton S.M., Vickers M.H. (2016). Realizing the Potential of Adolescence to Prevent Transgenerational Conditioning of Noncommunicable Disease Risk: Multi-Sectoral Design Frameworks. Healthcare.

